# A new pH sensitive fluorescent and white light emissive material through controlled intermolecular charge transfer†
†Electronic supplementary information (ESI) available: Text: experiments, characterization, devices fabrication. Figures: NMR, titration curve, estimation of p*K*_a_, single quantum mode fit, absorption/PL spectrum in pH 2/12, different orbitals for molecule **A**, half-occupied natural orbitals, PL spectra of **ATAOPV** at different concentrations under different pH, experimental and calculated absorption and emission. See DOI: 10.1039/c4sc01911c



**DOI:** 10.1039/c4sc01911c

**Published:** 2014-09-10

**Authors:** Y. I. Park, O. Postupna, A. Zhugayevych, H. Shin, Y.-S. Park, B. Kim, H.-J. Yen, P. Cheruku, J. S. Martinez, J. W. Park, S. Tretiak, H.-L. Wang

**Affiliations:** a Physical Chemistry and Applied Spectroscopy (C-PCS) , Chemistry Division , Los Alamos National Laboratory , Los Alamos , New Mexico 87545 , USA . Email: hwang@lanl.gov; b Theoretical Division , Los Alamos National Laboratory , Los Alamos , New Mexico 87545 , USA . Email: serg@lanl.gov; c Department of Chemistry/Display Research Center , Catholic University of Korea , Bucheon 420-743 , Republic of Korea; d Center for Integrated Nanotechnologies , Materials Physics and Applications Division , Los Alamos National Laboratory , Los Alamos , New Mexico 87545 , USA

## Abstract

A new, pH dependent and water-soluble, conjugated oligomer (amino, trimethylammonium oligophenylene vinylene, **ATAOPV**) was synthesized with a quaternary ammonium salt and an aromatic amine at the two ends of a π-conjugated oligomer, thus creating a strong dipole across the molecule. A unique white light LED is successfully fabricated from a stimuli responsive organic molecule whose emission properties are dominated by the pH value of the solution through controlled intermolecular charge transfer.

## Abstract 2

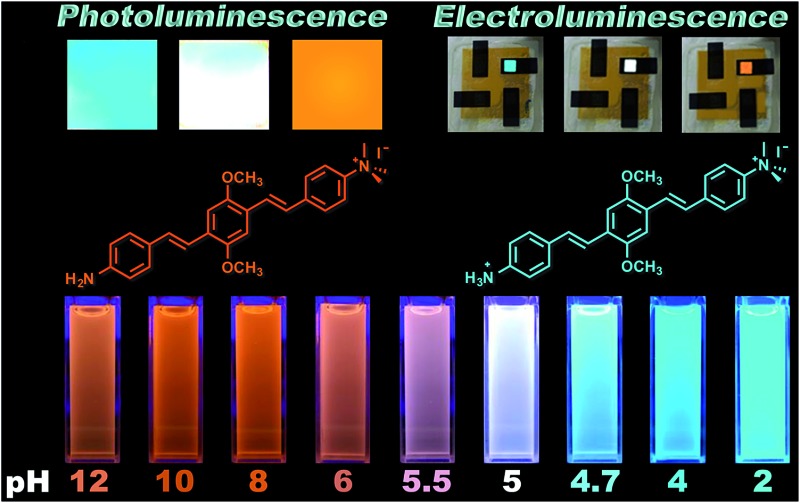
Fabrication of a unique white light LED from a stimuli-responsive organic molecule is reported. Emission properties are dominated by the pH of the solution through intermolecular charge transfer.

## Introduction

Conjugated materials have been extensively studied in recent years due to their tunable physical, optical and electronic properties and their applications in optoelectronics^1^ and bio-sensor technologies.^2^ Of particular interest are the *p*-phenylene vinylene (PPV) derivatives, one of the most extensively studied conjugated materials due to their high quantum yield and tunable structures that lead to desired physical and optical properties.^3^ There is a long history of developing pH sensitive chromophores for biomedical research. In recent years, the development of pH-dependent conjugated chromophores has shown promise for the bio-imaging of living cells^4^ and sensing of proteins^5^ and DNA.^6^ Previously reported pH-dependent organic materials are xanthene,^7^ cyanine,^8^ fluorene,^4^ acridine^9^ and BODIPY^4^ derivatives that show strong pH dependent fluorescence properties. Among all these chromophores, xanthene derivatives are of special interest as they exhibit white light emission under specific pH values;^7^ specifically, Huynh *et al.* recently synthesized a dithienophosphole derivative that exhibits white light emission from a single molecule species.^10^ Despite this recent example, there has been limited success in developing a white emissive material resulting from a single molecule,^11^ because the majority of molecular materials obey Kasha's rule, exhibiting single-band fluorescence from the lowest singlet excited state.^12^ In addition, even when light emission is generated due to a mix of multiple fluorescence peaks, there is a tendency towards energy cascading from high to low energy, which leads to a dominant emission color corresponding to the lowest energy. To overcome this low energy emission, several approaches have been attempted including those employing excited-state intramolecular proton transfer (ESIPT),^13^ excimers,^14^ and isomers in different pHs.^10^

In this manuscript we report the synthesis and characterization of a conjugated oligomer (**ATAOPV**) with pH-dependent fluorescence properties. We show that at pH 5.0, this oligomer exhibits dual fluorescence of blue and red light leading to white light emission in aqueous solution, solid films and OLED devices. Our conjugated oligomer, **ATAOPV**, with a strong permanent molecular dipole moment, is unique as we can control (turn on/off) the excited state charge transfer *via* protonation/deprotonation. In addition to the white light emission, this **ATAOPV** can produce blue and orange emissions, tuned by pH. Our electronic structure modeling results suggest that the lower energy peak, formed at high pH values, is primarily attributed to intermolecular charge transfer. We have successfully fabricated LED devices that emit blue, orange or white light. Although the yield of the LED is relatively low, our study reveals a unique white light LED fabricated from a stimuli responsive organic molecule whose emission properties are dominated by the pH value of the solution through controlled intermolecular charge transfer.

## Methods

### Synthesis

**Scheme 1:**
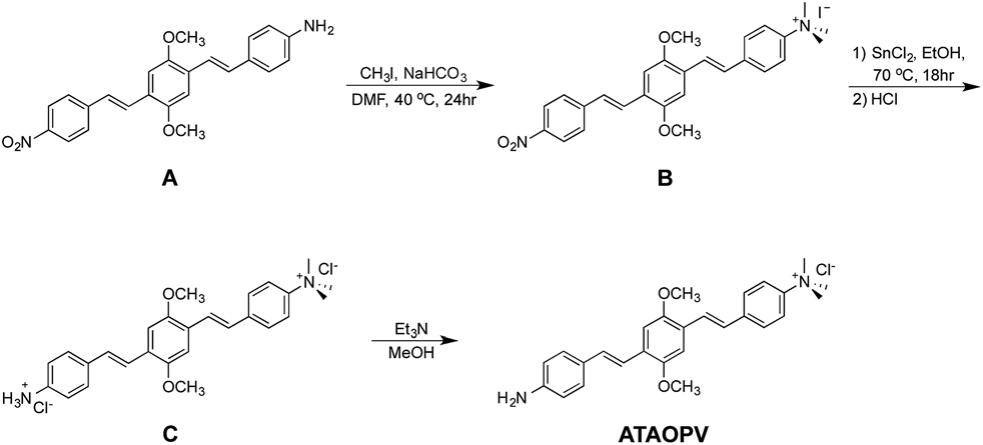
Synthetic scheme for **ATAOPV**.

The reaction scheme of **ATAOPV** is shown in Scheme 1. Compound **A** is synthesized by mono-reduction of the dinitro-OPV compound,^15^ which is achieved using a procedure that involved chloromethylation of 1,4-dimethoxybenzene, converted into the phosphonate and then coupled with a vinyl group *via* Horner–Wadsworth–Emmons reactions.^3^ The reduction of the nitro group into the amine was achieved by reacting dinitro-PPV with pyridine and sodium sulphate under relatively mild reaction conditions. Further treatment of the product with an excess of methyl iodide leads to formation of the quaternary ammonium group. The remaining nitro group is then converted to the amine using a strong reducing agent, stannous chloride in acidic conditions. The end product has a protonated amine salt which is then neutralized to the amine using triethylamine in methanol solution. The as-synthesized PPV oligomer is then purified *via* recrystallization resulting in a pure yellow solid. The ^1^H and ^13^C NMR spectra of **ATAOPV** are illustrated in Fig. S1† and agree well with the proposed molecular structures. Detailed information on synthesis, characterization, and OLED fabrication is provided in the ESI.†

### Computational methods

We performed quantum-chemical modeling of protonated and non-protonated molecules as well as their dimer structures. The CAM-B3LYP density functional^16^ combined with the 6-31G* basis set was used as a default method for all molecular modeling, carried out using the Gaussian 09 program.^17^ Density Functional Theory (DFT) and time-dependent DFT (TDDFT) frameworks^18^ have been used for calculations of ground and excited state electronic properties, respectively. CAM-B3LYP is a long-range corrected hybrid functional providing a correct description of neutral electronic excitations and charged states of extended π-conjugated systems.^19^ Because excitation energies are highly sensitive to the choice of density functional, performance of CAM-B3LYP was compared to that of B3LYP and ωB97X functionals (see ESI).† The 6-31G* basis set is known to provide adequate results for geometry and energy values for relatively large molecules consisting of the first and second row elements,^20^ in a noticeably short CPU time compared to that of larger basis sets. Notably, the inclusion of polarization orbitals is important for excited states and charged molecules.^18^ The introduction of diffuse orbitals is known to enhance the accuracy of the acidity prediction,^18^ however, for the given system the improvement is found to be insignificant.

Frontier orbitals (including HOMO and LUMO), natural orbitals (NO), and natural transition orbitals (NTO)^21^ are used for visual analysis of the electronic wave function of the ground and excited states. Natural (transition) orbitals give the best representation of a one-electron (transition) density matrix in terms of molecular orbitals, irrespective of the quantum chemistry method by which that density is obtained. Consequently, a comparison of NO/NTO with the frontier orbitals allow for a robust identification of the nature of the excited/anionic/cationic states, provided that the occupation numbers of the natural orbitals are close to integers (which is true for all of the considered molecules/states). In particular, for an anion/cation we look at the half-occupied NO; the occupation numbers of the two adjacent NOs should be close to 2 and 0 in order for such NO analysis to be meaningful. In the NTO analysis of an excitation transition we look at the highest occupied (hole NTO) and lowest unoccupied (electron NTO) orbitals, whose NTO weights should be close to 1 to identify such transitions with a single excitation. In the NO analysis of an excited state corresponding to a single excitation, we look for a pair of orbitals at the boundary between “doubly occupied” and “vacant” NOs. The difference in occupations of these two NOs characterizes the amount of charge transfer in the excited state, whereas the spatial separation of the corresponding hole and electron NTOs characterizes the charge separation itself.

To implicitly account for the influence of the media on the investigated properties, the polarizable continuum model (PCM) with the appropriate static and optical dielectric constants for water has been used for geometry optimization and calculation of optical spectra. Solvation of excited states is simulated using the state-specific (SS) approach.^22^ An alternative, linear response (LR) method^23^ leads to nearly the same result for the first absorption peak. Calculation of the acid dissociation constant (see details in ESI†) is carried out using the SMD solvation model,^24^ which also includes non-electrostatic solvation effects. This model is the recommended choice for computing Gibbs free energy of solvation, due to its fine-tuned parameters.^17^ Explicit solvation was used in classical molecular dynamics (MD) simulations with the MM3 force field.^25^ These calculations are carried out using the TINKER program.^26^ The MM3 water box has been obtained by annealing the TIP3P water box. The simulations are conducted at 400 K (at 300 K the thermodynamic properties of MM3-water do not match experiment, as it is a high-density liquid).

In molecular calculations, counter-ions that could possibly be present in a solvent environment have been neglected, and the groups NMe_3_ and NH_3_ are assumed to be positively charged in solution. This assumption is tested as follows. First, the only stable position of the iodide bound to the non-protonated molecule is in the NMe_3_^+^ “pocket”. Then the kinetic stability of this configuration has been tested by MD in water: at 400 K the iodide dissociates from the molecule in tens of picoseconds. Finally, even if iodide is placed in the “pocket”, its direct influence on the absorption/emission spectra as well as on the direct electron transfer is negligible, see Table S1.†

Ground state molecular geometries have been optimized in vacuum and water. In both cases the geometries are topologically equivalent to each other. The lowest excited state structures have been subsequently optimized starting from the ground state geometry in the corresponding media. The π-conjugated system is planar in the excited state and non-planar in the ground state, with the terminal benzene rings slightly rotated around the long axis of a molecule, in agreement with previous studies.^27^ Only the most energetically favorable molecular conformation is considered here, since it was shown previously^3^ that the difference in the optical properties of various conformations is insignificant. Selected dimer configurations are obtained by π-stacking two molecules at 3.5 Å with zero slip-stack displacement in the orientation minimizing electrostatic and steric interactions. Dimer geometry in the ground state has been optimized by using the ωB97X functional, which accounts for dispersive interactions. Finally, statistical sampling of dimer geometries is performed by 10 ns MD in a water box using the MM3 force field.

## Results and discussion

### Observed pH-dependent optical properties

**Fig. 1:**
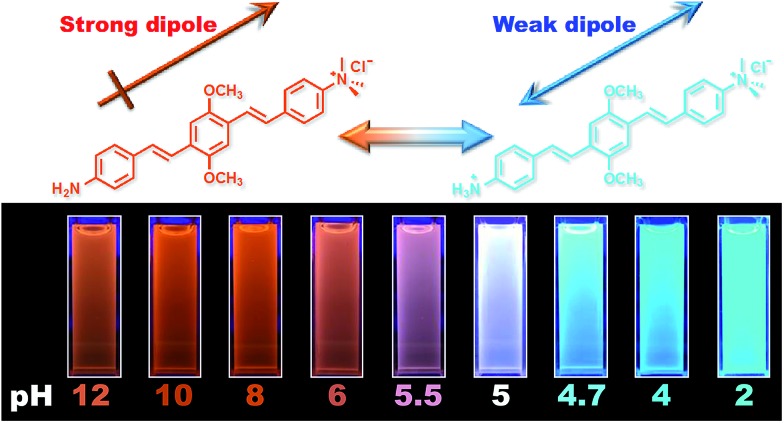
(Top) Diagram of the protonation of **ATAOPV**: on the left is the non-protonated molecule whose chemical formula is abbreviated as NH_2_-OPV-NMe_3_^+^, on the right is the protonated molecule NH_3_^+^-OPV-NMe_3_^+^. (Bottom) Photographs of **ATAOPV** multicolor emission as a function of pH 12–2 in solution (10^–5^ M in H_2_O).

Fig. 1 shows the chemical structures of non-protonated and protonated **ATAOPV** molecules (top panels) and their emission properties in aqueous solution at various pHs (bottom panel). The fluorescence color of **ATAOPV** spanning from red to blue is associated with the decrease in pH of the aqueous solution from 12 to 2. Judging from the titration curve (Fig. S2†), we can reasonably assume that the fluorescence at pH 12 and pH 2 originates from the non-protonated and protonated molecular species, respectively. It is interesting to note that white light emission is attained when the pH value reaches 5.0.

**Fig. 2:**
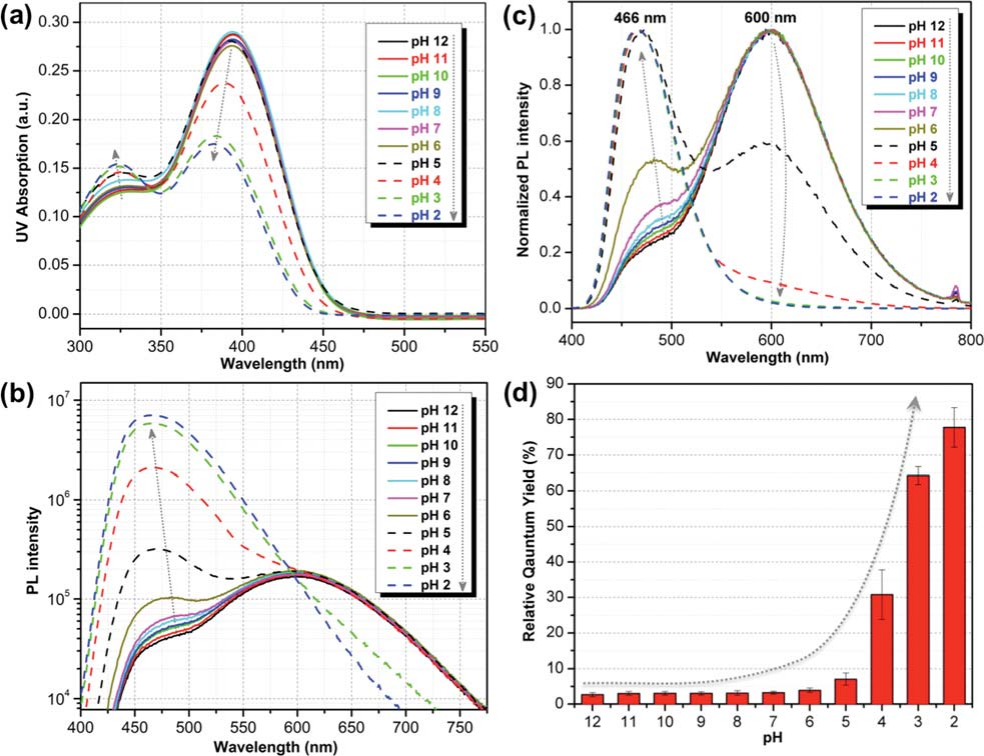
pH-dependent optical properties of **ATAOPV** (10^–5^ M in H_2_O): (a) UV-vis absorption spectra; (b) normalized PL spectra; (c) absolute PL spectra in logarithmic scale; (d) relative quantum yield using diphenylanthracene (DPA) in ethanol, as standard.

The optical properties of **ATAOPV** in water (10^–5^ M), as a function of pH, are summarized in Fig. 2. The UV-vis absorption of the aqueous solution of **ATAOPV** at pH 12 shows two distinct peaks at 324 nm and 394 nm (Fig. 2a). Decreasing the pH leads to a blue shift of the absorption peak (from 394 nm to 384 nm) and a concomitant decrease of the peak intensity. The photoluminescence (PL) spectra of **ATAOPV** (Fig. 2b and c) generally have two broad peaks at 466 nm and 600 nm, which are particularly pronounced at pH 5 resulting in white light emission. The absolute intensity of the 466 nm peak increases dramatically with decreasing pH (Fig. 2c). The measured quantum yield (QY) decreases from 77.8% to 2.6% as the pH increases from 2 to 12 (Fig. 2d). Such a dramatic decrease in the quantum yield accompanied by the huge PL-redshift suggests a charge transfer (CT) character of the lowest excited state of the non-protonated **ATAOPV**.^13a,28^

Statistical analysis of the pH-dependence of the absorption and emission spectra (see ESI† page S9) confirms that these spectra can be represented as a linear superposition of the spectra of two species whose relative concentration is described by the formula:1
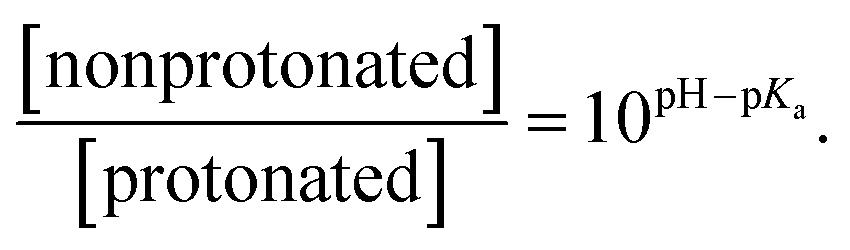



**Fig. 3:**
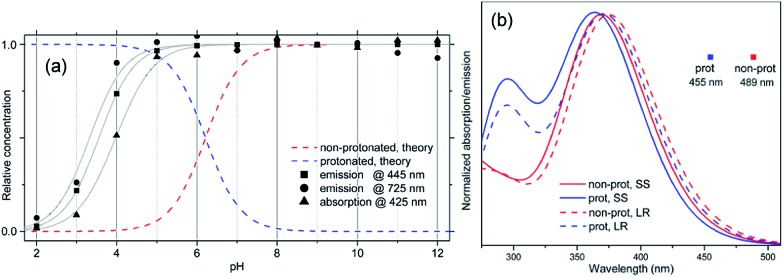
(a) Calculated dependence of the concentration of protonated and non-protonated species on the pH of the solution, compared to data extracted from the experimental absorption spectrum at 425 nm, and emission spectrum at 445 and 725 nm. Gray curves are obtained by fitting eqn (1) to these data. (b) Calculated absorption spectra and PL peak position for protonated and non-protonated molecules in water. In SS calculations only the first state was optimized. Spectral line shapes are obtained by Gaussian broadening of vertical electronic transitions.

From this analysis, the estimated p*K*_a_ is 3.3–3.6 from the PL spectra and 3.6–4.0 from the absorption spectra (Fig. S3†). These results are consistent with the titration curve (Fig. S2†), which gives the upper limit for the p*K*_a_ at roughly 4.0. The *ab initio* estimate for the p*K*_a_ is 6.2, so the calculated protonation energy of 0.36 eV is overestimated by 0.15 eV, probably due to the use of an implicit solvation model in our simulations. In any case, both experiment and theory suggest that protonated molecules are the dominant species at pH 2, whereas at pH 12 their concentration is negligible (Fig. 3a).

A deeper insight can be obtained by analyzing the shape of the absorption and emission peaks. The results of such analysis (Fig. S4†) demonstrate that absorption spectra at pH 2 and pH 12 can be fitted by a Gaussian broadened vibronic progression, with the parameters typical for functionalized OPV molecules.^19*a*^ The emission spectra are structureless, with less broadening than that for absorption. At pH 2, the blue PL band has a single component, whereas at pH 12 there are two Gaussian bands: red and blue. The obtained vertical excitation energies and Stokes shifts are summarized in Table 1.

**Table 1:** Experimental and calculated absorption energy and Stokes shift corresponding to vertical electronic transitions. The “reference” molecule is NMe_3_^+^-OPV-NMe_3_^+^ in water. At pH 12 two emission components are given: blue and red. For the first three rows the theoretical calculations are performed for a single molecule in water (SS solvation). In the last row the emission energy is calculated as *E*(+1,+1) – *E*(0,+1) + *E*(–1,–1) – *E*(0,–1), where *E*(*Q*_1_,*Q*_2_) is the energy of the molecule with extra charge *Q*_1_ in the geometry optimized with charge *Q*_2_ (dimer approximated as infinitely separated cation and anion). An extended version of this table is given in the ESI (Table S1)

	Absorption, eV		Stokes shift, eV	
	Exp.	Theory	Exp.	Theory
Reference	3.21	3.30	0.68	0.52
pH 2	3.26	3.32	0.68	0.54
pH 12	3.16	3.28	0.63	0.66
pH 12 red			1.17	1.16

**Fig. 4:**
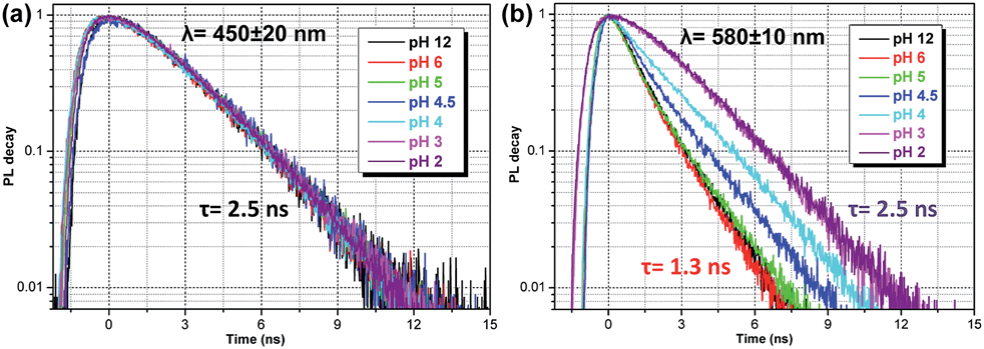
Fluorescence lifetime decay curves of **ATAOPV** (10^–5^ M in H_2_O) as a function of pH in two spectral windows: (a) 450 ± 20 nm and (b) 580 ± 10 nm. The excitation wavelength is 405 nm.

The time-resolved fluorescence decay lifetime measurements (Fig. 4) conducted separately in the blue (450 ± 20 nm) and red (580 ± 10 nm) spectral regions confirm that there are two emission components with distinct lifetimes (1.3 and 2.5 ns) and, consequently, there is no efficient energy transfer between them. Assuming the Forster type energy transfer to be the only energy exchange channel between molecular species, experimental observations across the entire pH range are then consistent with the presence of two molecular species with negligible energy transfer. The latter is rationalized by vanishing spectral overlap between the absorption at pH 12 (394 nm, non-protonated species) and emission at pH 2 (466 nm, protonated species, see Fig. S5†).^13*a*^

Measured PL lifetimes (*τ*) and QY allow for estimation of the radiative and non-radiative times using the formulas

2 *τ*_rad_ = *τ*/IQY, *τ*_nrad_ = *τ*/(1 – IQY),

where IQY is the internal QY. If IQY is close to QY then the calculated times are *τ*_rad_ = 2.5–3 ns, *τ*_nrad_ ≥ 10 ns at 466 nm, and *τ*_rad_ ≥ 50 ns, *τ*_nrad_ = 1.3 ns at 600 nm. The radiative lifetime at 466 nm corresponds to the oscillator strength ∼1, which is typical for a single OPV molecule emission.^3^ The radiative lifetime at 600 nm corresponds to the oscillator strength ∼0.1, which may be a sign of a weakly emitting CT state. It should be noted that the kinetics of PL decay at 600 nm for pH 12 is not single exponential: at long times the slope of PL decay approaches that for pH 2 (Fig. 4b), which, by magnitude, is consistent with the tails of the blue PL band in Fig. 2c.

To summarize, the experimental observations are fully consistent with the assumption that at pH 2, the optical properties are dominated by the protonated highly emissive **ATAOPV** molecules in a dilute aqueous solution, whereas at pH 12, two distinct non-protonated species are present: one with optical properties similar to the protonated molecule (very minor concentration) and another one (dominant concentration) having the CT lowest excited state. To elaborate on this hypothesis and determine the nature of the CT state we have performed first principle modeling of molecules and their aggregates.^29^

### *Ab initio* calculations of single-molecule absorption and emission spectra

Compared to substitutions with neutral donor–acceptor groups, the influence of NMe_3_^+^ and NH_3_^+^ charged groups on the electronic properties of the functionalized OPV molecule has important differences. This can be rationalized by examining various orbital plots. In particular, strong acceptors such as NO_2_ (see Fig. S6†) influence the host molecule mainly by hybridizing its low-energy π-system with that of the OPV molecule, whereas the effect of creating an extra electric field for the π-system is less important.^19*a*^ In contrast, the charged groups create a much stronger electric field, whereas the sp^3^ hybridization of the nitrogen atom pushes its frontier π-orbitals out of resonance with the π-system of the host molecule. That is why wave-functions of the molecules functionalized by NMe_3_^+^ and NH_3_^+^ groups are essentially the same as that for the molecules with hydrogen in place of these groups, *i.e.* the π-isoelectronic molecules OPV-NH_2_ and OPV (see Fig. S7–S10†). At the same time, the energetic characteristics, which are sensitive to charge and electric field distribution, such as solvatochromic shift and CT state transition energy, are different (Fig. S7†). These considerations help to interpret the first-principle calculations results presented below.

**Fig. 5:**
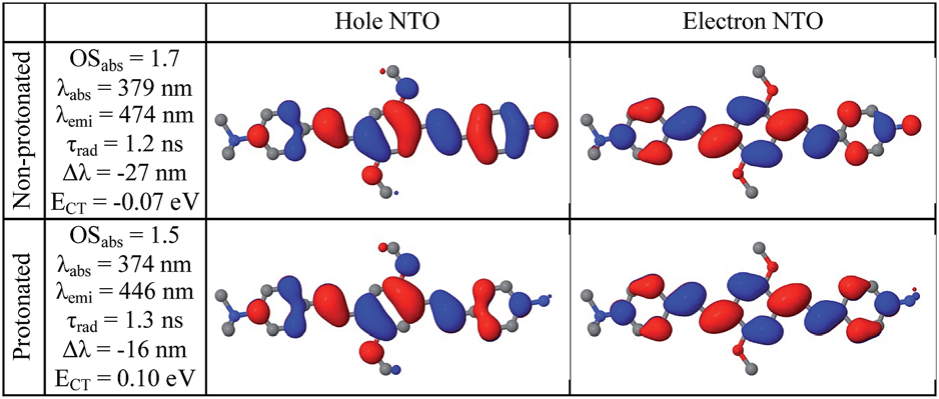
Calculated properties of the first excited state in water of protonated and non-protonated molecules. Natural transition orbitals are shown for the lowest excitation in the relaxed geometry. OS is the oscillator strength, *τ*_rad_ is the radiative lifetime, Δ*λ* = *λ*_emi_(water) – *λ*_emi_(vacuum) is the solvatochromic shift, *E*_CT_ = *E*_cation_ + *E*_anion_ – *E*_exciton_ – *E*_ground_ is the intermolecular CT energy relative to the intramolecular exciton (relaxed geometries, equilibrium solvation).

Calculated single-molecule absorption spectra are in good agreement with experiment (Fig. 3b and Table 1). The main peak is well resolved and is composed of a single electronic transition. NTO analysis (Fig. 5) clearly identifies it as a HOMO to LUMO π–π* transition of the conjugated system with a large transition dipole directed along the molecule. The pH dependence of both the peak position and its intensity is qualitatively reproduced by calculations (quantitatively, theory underestimates the changes) as shown in Fig. 3a. The second absorption band is comprised of several competing electronic transitions. The more pronounced character of this band for the protonated molecules compared to the non-protonated species is well reproduced by calculations (compare Fig. 2a and 3b). All of the above features exactly replicate the corresponding trends for the π-isoelectronic molecules OPV and OPV-NH_2_, respectively.^3^

Calculated single-molecule emission peak positions and fluorescence lifetimes (Table 1 and Fig. 5) match the observed characteristics of the blue PL band (for both pH 2 and pH 12). Therefore, we can associate the blue PL band at pH 2 and pH 12 with the emission from protonated and non-protonated single molecules, respectively. Strictly speaking, the true origin of the blue PL band at pH 12 is unclear, because such a weak signal can also be produced by contaminants such as residual protonated molecules (which should be absent at pH 12).

The red PL band is presumably attributed to a CT state because of a large fluorescence lifetime, low QY, and strong solvatochromism. Restricted and unrestricted TDDFT calculations show no intramolecular CT state for any of the tested geometries: ground, excited, or hypothetical forced CT state (in the latter, opposite sides of a molecule are optimized with opposite extra charges), as well as for the lowest triplet state. The measured PL spectrum in tetrahydrofuran (Table S1†) also rules out the existence of an intramolecular CT state: the experiment shows positive solvatochromism, *i.e.* stabilization in a solvent, whereas the putative CT state should be destabilized in a more polar solvent because of the screening of the NMe_3_^+^ charge (for the same reason the non-CT state also shows negative solvatochromism). An intramolecular non-CT state on a charged molecule (electron transfer from the counter-ion) is also excluded because such a molecule would emit at 800 nm. Therefore, the experimentally observed red PL band has been attributed to an intermolecular CT state.

### Origin of the red PL band

There are at least three possible donor–acceptor complexes able to host an intermolecular CT state: a dimer of two molecules, a molecule + water, and a molecule + counter-ion. A rigorous theoretical study of each of these possibilities requires an explicit solvation of CT states as well as proper consideration of electronic processes in water, which themselves are challenging theoretical problems.^30^ For this reason we limit our investigation to the analysis of only the structural and energetic feasibility of a CT state. First, the prerequisite for an efficient CT formation is an availability of the ground state configuration of the donor–acceptor complex. Second, since the Coulomb interaction stabilizes the CT state, the energy of two infinitely separated charges gives a robust upper estimate of the lowest CT state energy.

**Fig. 6:**
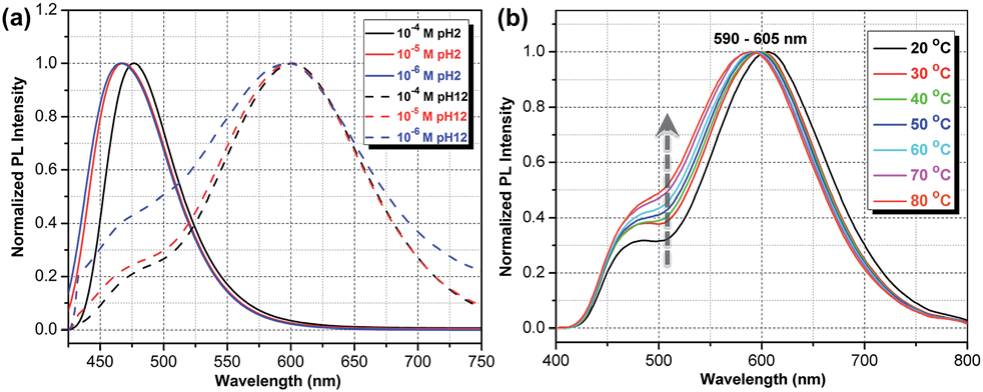
(a) Experimental PL spectra normalized in such a way that the maximum PL intensity at pH 2 equals 1, whereas the spectra at pH 12 are additionally scaled up by a fixed constant. (b) Temperature-dependent PL spectra in a 10^–5^ M **ATAOPV** aqueous solution reveal an increase in PL intensity of the blue emission with increasing temperature.

We start by considering an intermolecular CT on the dimer, which is known to produce a large solvatochromic shift *e.g.* for merocyanine dyes.^31^ Important parameters here are the ionization potential (IP) and electron affinity (EA) as well as exciton energy for non-protonated molecules in water. These 3 quantities calculated for respective vibrationally relaxed species are 4.8 eV, 2.2 eV, and 2.7 eV, respectively. We immediately see that the CT state on a dimer is lower in energy than the intramolecular exciton by 0.1 eV (see Tables 1 and S1†). The binding energy of a dimer in its ground state is about 0.5 eV as obtained in both *ab initio* ωB97X/6-31G* and MM3 force field calculations. Classical MD simulations in water at 400 K show that the dimer is stable for at least 10 ns, though its geometry fluctuates substantially. At large charge separation the estimated emission peak position exactly fits the observed red PL band (Table 1). Calculations for selected bound dimer configurations show essentially the same red-shifted emission (Table S1†) as well as giving very weak oscillator strengths consistent with the observed radiative lifetimes. At the same time, the optical absorption spectrum of a dimer should not deviate from the single-molecule absorption because of large geometry fluctuations suppressing delocalized intermolecular excitations. The dimer picture is also consistent with the observed decrease of PL intensity with dilution relative to the blue emission band (see Fig. 6a and S11†). For protonated species the intermolecular CT state is energetically unfavorable; in addition, there are no stable dimer configurations in the ground state.

For the charge transfer to solvent scenario there are two options. The variant with the non-protonated molecule being the acceptor is energetically unfavorable because the IP of liquid water is large, at about 6.5 eV.^32^ That IP is not exactly the energy of the solvated cation (H_2_O^+^ or H_3_O^+^) but corresponds to the light-driven electron detachment. The EA of water is 3–4 eV (ref. 33) making the electron transfer to water energetically feasible in the form of a bulk or surface solvated electron. The solvated electron has a short lifetime, hundreds of picoseconds,^33*b*^ and allows for plenty of nonradiative recombination channels,^30*a*^ that is consistent with the low QY of red PL. As the dispersion of energies of the solvated electron, 0.5 eV,^33b,c^ is much larger than the observed emission bandwidth, 0.2 eV, this kind of charge transfer determines mainly the nonradiative channel. In contrast to the non-protonated molecule, the protonated one has a higher IP by 0.5 eV and a very different solvation shell that may suppress the charge escape to the solvent.

Finally, the charge transfer to iodide can be excluded: its IP/EA is too high/low. Also we did not find any electronically coupled configurations of the molecule + iodide complex similar to that observed for other systems.^34^ In summary, the intermolecular CT state is structurally and energetically feasible in two scenarios: dimer and charge transfer to the solvent, with the former being the primary radiative channel and the latter being the mainly nonradiative channel.

To further test the dimer model, the temperature dependence of the PL spectra was measured (Fig. 6b). The observed increase in the relative intensity of the blue emission component, with temperature increases, is consistent with the increased thermal dissociation of dimers. At the same time, this trend is inconsistent with the intramolecular CT hypothesis: the relaxation of initial high-energy excitation to a putative low-lying intramolecular CT state should be faster at higher temperatures, yielding the opposite temperature behavior of the blue-to-red PL intensity ratio. Also, the observed negative thermochromism of the red emission peak has no simple explanation in an intramolecular CT model, whereas in the dimer model it is a consequence of the increased intermolecular separation leading to an upshift of the lowest excitation energy.

### White emission and electroluminescence

**Fig. 7:**
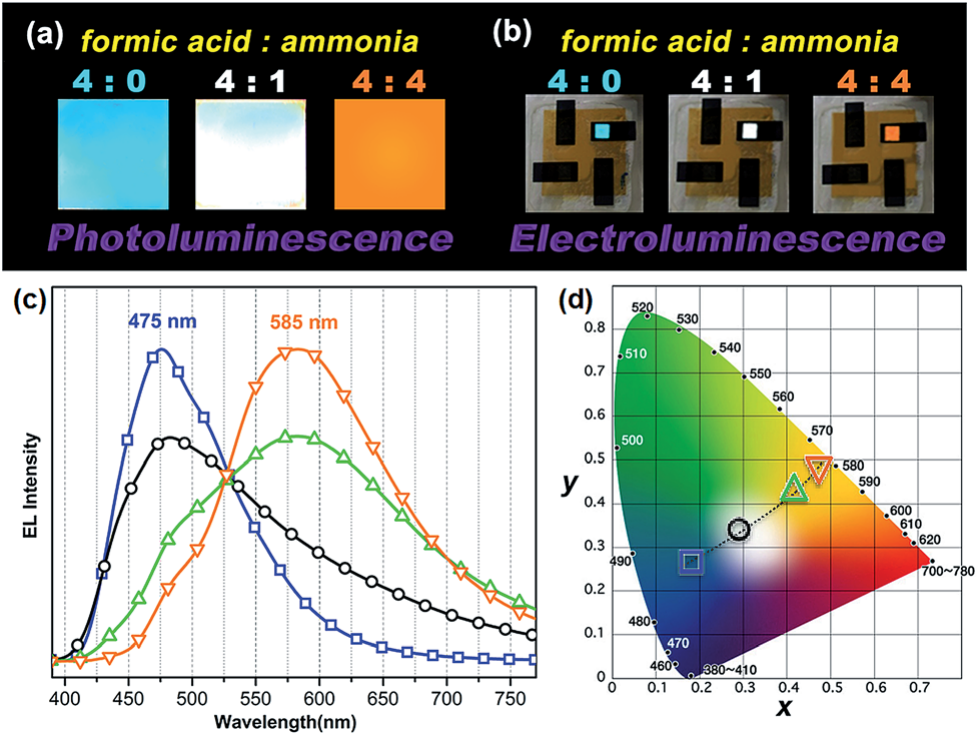
(a) Photographs of thin film emission dependent on pH under a UV lamp and (b) the image of LED devices under operation. (c) EL spectra and (d) CIE chromaticity diagram dependent on formic acid to ammonia ratio (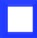) 4 : 0, (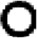) 4 : 1, (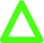) 4 : 2, and (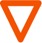) 4 : 4.

**Fig. 8:**
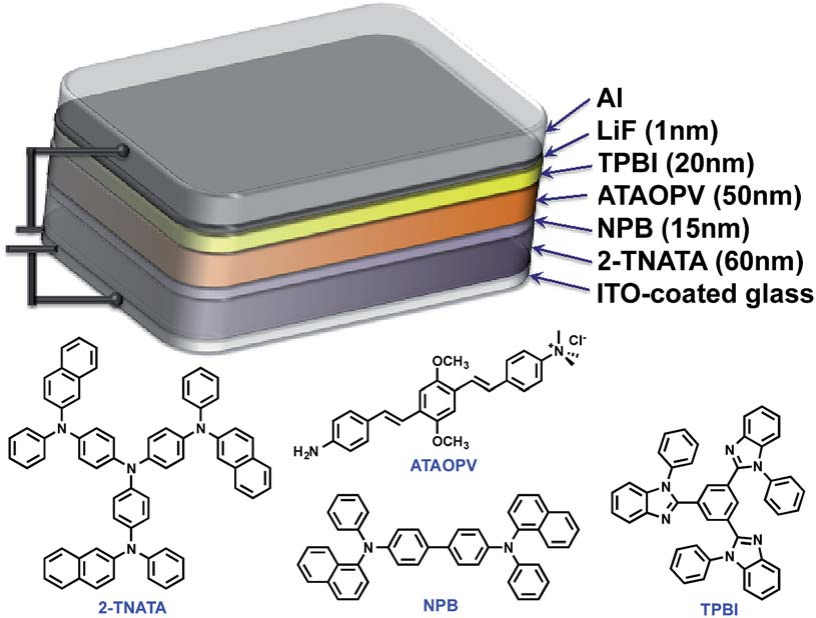
Schematic illustration of OLED structure.

One very interesting property of **ATAOPV** in the pH 5 aqueous solution is the white light emission resulting from the combination of two emission colors at 466 and 600 nm. The pH-dependent optical properties in solution are also reflected in films and organic light-emitting diodes (OLEDs) fabricated by spin-coating from the **ATAOPV** solution. As shown in Fig. 7a, the films from different pH solutions are shown as blue, white, and orange under UV excitation. In OLED devices, the as-synthesized **ATAOPV** was used as an emitting material layer (EML). The EMLs were spin-coated from 1 wt% of **ATAOPV** solutions with different ratios of formic acid (acid) : ammonia (base) (4 : 0, 4 : 1, 4 : 2, 4 : 4) and the spin-coated thin films were dried overnight in a 110 °C vacuum oven to ensure complete evaporation of formic acid and ammonia. The other layers were vacuum deposited with device architectures that include a hole inject/transport layer, 4,4′,4′′-tris-(*N*-(naphthylen-2-yl)-*N*-phenylamine)triphenylamine (2-TNATA), *N*,*N*′-di-[((1-naphthyl)-*N*,*N*′-diphenyl]-1,1′-biphenyl)-4,4′-diamine (NPB), and a hole blocking/electron transporting layer, 1,3,5-tris(*N*-phenylbenzimidizol-2-yl)benzene (TPBI). The complete multilayered device architecture is indium tin oxide (ITO)/2-TNATA/NPB/**ATAOPV**/TPBI/LiF/Al (Fig. 8). Electroluminescence (EL) spectra (colors) of LED devices are determined by the formic acid and ammonia (base) ratios (Fig. 7b and c). The shift in the EL spectra corresponds to the change in the CIE chromaticity diagram coordinates from (0.18, 0.27) to (0.46, 0.48) (see Fig. 7d). Overall, this trend in the EL spectra is in good agreement with the shifts from the blue to red emission regions observed in the PL spectra of the **ATAOPV** in solution, as a function of pH. Of particular interest is the white emission EL device as CIE (0.28, 0.34) with a color rendering index (CRI) of 81.9 and the colors of the EL emission match very nicely to that of the thin film under UV excitation (see Fig. 7a and b). The turn-on voltage (*V*) and external quantum efficiency, EQE (%) for the EL devices are 3.0 V (0.08%), 2.25 V (0.001%) and 4.5 V (0.007%) for blue, white, and orange EL devices, respectively. The efficiency of these LED devices is low mainly because of the ionic nature of the emitter. The ionic species lead to shallow or deep traps inside the HOMO and LUMO levels that quench excitons.^35^ Although the EQE for the white light LED is relatively low, research to optimize the device performances is ongoing. Altogether, these results from solution, film and device are experimental demonstrations of multi-color emission through a pH-controlled interplay of intra- and inter-molecular fluorescent molecular species.

## Conclusions

We have designed and synthesized a water-soluble oligomer **ATAOPV** with amine (electron donor) and ammonium salt (electron acceptor) on opposite ends of the molecule. This molecule shows a dramatic change in fluorescence color from red to blue (bottom panel in Fig. 1) and a 30 fold increase in quantum yield from 2.6 to 77.8% as the pH decreases from 12 to 2 coupled with a gradual change from the non-protonated to protonated molecular species (top panel in Fig. 1). Both protonated and non-protonated species have absorption spectra over a broad range from 300 to 450 nm. Our TDDFT quantum chemical simulations attribute this absorbance to π–π* intramolecular transitions of the conjugated system. Likewise, the blue band appearing in the PL spectra is assigned to intramolecular emissions of protonated and non-protonated molecules, as suggested by theoretical modeling and fits of time-resolved emission dynamics. In contrast, the red PL band appearing with increasing concentration of non-protonated molecules is suggestive of the appearance of a weakly emissive intermolecular CT transition. In particular, this red PL dominates the fluorescence at pH 12. Our simulations ascribe this red PL to a CT state in an antiparallel dimer of non-protonated molecules (determining mainly the radiative decay channel) and electron transfer to solvent (determining mainly the non-radiative decay channel). Such emissive properties of **ATAOPV** are unique and different compared to the intramolecular CT transitions observed in many previously reported conjugated molecules with strong donor and acceptor substituents.^36^ At pH 5.0, **ATAOPV** in aqueous solution exhibits dual color emission originating from both blue and red broad fluorescence bands. Unique emissive properties of **ATAOPV** have been further used to fabricate thin-film LEDs displaying blue, white and orange colors as we vary the processing conditions (changing the ratio between formic acid and ammonium hydroxide). Specifically, the LED spectra variations correspond to the CIE coordinate change from (0.18, 0.27; blue) to (0.28, 0.34; white) to (0.46, 0.48; orange). Thus our joint experimental and theoretical study suggests a pH controlled ratio of protonated and non-protonated species leading to tunable fluorescence across the entire visible region. Our experiments demonstrate multi-color emission resulting from a single molecular species in solution, thin film and LED device, which may have implications for light-emitting technologies based on organic semiconductor materials.

## Abbreviations

CT: Charge transfer
HOMO: Highest occupied MO
LR: Linear response
LUMO: Lowest unoccupied MO
MD: Molecular dynamics
MO: Molecular orbital
NO: Natural orbital
OLED: Organic light-emitting diode
PL: Photoluminescence
QY: Quantum yield
SS: State specific
TDDFT: Time-dependent density functional theory
