# Supplementary material for: A new pH sensitive fluorescent and white light emissive material through controlled intermolecular charge transfer
Source: Chem Sci. 2014 Sep 10;6(1):789–97. doi: 10.1039/c4sc01911c (PMC5592806; doi:10.1039/c4sc01911c)
Supplement: Supplementary file 1 [file SC-006-C4SC01911C-s001.pdf]

Electronic Supplementary Material (ESI) for Chemical Science.
This journal is © The Royal Society of Chemistry 2014

Supporting Information

A New pH Sensitive Fluorescent and White Light Emission Material through Controlled Intermolecular Charge Transfer

Young-Il Park,[a,¥] Olena Postupna,[b,¥] Andriy Zhugayevych,[b,¥]
Hwangyu Shin,[c] Young-Shin Park,[a] Beomjin Kim,[c] Hung-Ju Yen,[a] P. Cheruku,[a]
Jennifer S. Martinez,[d] Jongwook Park,[c] Sergei Tretiak,[b]* Hsing-Lin Wang[a]*

[a] C-PCS, Chemistry Division, Los Alamos National Laboratory, Los Alamos, New Mexico, 87545

[b] Theoretical Division, Los Alamos National Laboratory, Los Alamos, New Mexico, 87545

[c] Department of Chemistry/Display Research Center, Catholic University of Korea, Bucheon 420-743, Republic of Korea

[d] Center for Integrated Nanotechnologies, Materials Physics and Applications Division, Los Alamos National Laboratory, Los Alamos, New Mexico, 87545

Contents

Experimental details: Characterization S2
Synthesis of **ATAOPV** S3
Figure S1. ^{1}H and ^{13}C NMR data of **ATAOPV** S5
Figure S2. HPLC chromatogram of **ATAOPV** S6
Figure S3. ESI-MS spectrum of **ATAOPV**. S7
Figure S4. Titration curve of **ATAOPV** S8
Figure S5. Estimation of pKa from absorption/emission spectra S9
Calculation of acid dissociation constant S10
Estimation of pKa from the experimental data S11
Figure S6. Single quantum mode fit to experimental spectra at pH 2 and pH 12 .... S12
Figure S7. UV/Vis absorption in pH 2 and PL spectrum in pH 12 .... S13
Figure S8. Comparison of different orbitals for molecule **A** in DMSO S14
Figure S9. Comparison of the studied molecules with π-isoelectronic analogues S15
Figure S10. Half-occupied natural orbitals of the lowest excited state S16
Figure S11. Half-occupied natural orbitals of the cation and anion S17
Figure S12. Comparison of different orbitals for non-protonated molecule in water S18
Figure S13. PL spectra of **ATAOPV** at different concentrations under different pH S19
Figure S14. PL spectra of ATAOPV in various solvents and solvent combinations.
Figure S15. Current density−voltage and luminance−voltage characteristics of (a) blue, (b) white, and (c) orange EL devices S20
Table S1. Experimental and calculated absorption and emission excitation energies S21

Experimental details: Characterization

All reagents and solvents are purchased from Aldrich or Fisher and used without further purification. ^{1}H and ^{13}C NMR spectra were recorded on a Bruker Avance 400 spectrometer. The IR spectra were recorded on a NICOLET-750 FT-IR spectrometer with KBr. Mass spectra were recorded on a Waters Synapt G2-S. HPLC analysis: Waters C18, 5 μm LC Column100×2mm, 400 nm (t = 0 - 5min, 70% A, 30% B; t = 5 - 30min, 50% A, 50% B, t=30 - 45min, 100% B. (A= water, 0.05% trifluoroacetic acid, B = methanol). Flow rate = 1 mL/min, tR = 21.52 min). The optical absorption and fluorescence spectra were obtained with Varian Cary 500 and Varian Cary Eclipse spectrometers. Quantum yields (QY) of **ATAOPV** solutions were measured using water as solvent and diphenylanthracene in ethanol as standard. The pH value of ATAOPV solution was controlled by 0.02 M NaOH and HCl solution.

Fabrication of OLED device

OLED devices were fabricated using ATAOPV as the emitting layer in the following structure: ITO/2-TNATA (60 nm)/NPB (15 nm)/ATAOPV (50 nm)/TPBI (20 nm)/LiF (1 nm)/Al, where 2-TNATA was used for the hole injection layer (HIL), NPB was the hole transport layer (HTL) and TPBI was the electron transport layer (ETL). An emitting material layer (EML) using ATAOPV were spin-coated at 1,000 rpm from 1 wt % with different ratio formic acid: ammonia (4:0, 4:1, 4:2, 4:4) solution and the spin-coated thin films were dried overnight in a 110 °C vacuum oven to ensure complete evaporation of formic acid and ammonia. The other organic layers were deposited under 10^{-6} Torr, with a rate of deposition of 1 Å/s to give an emitting area of 4 mm^2. The light intensity of EL device was obtained with a Minolta CS-1000A and a Keithley 2400 electrometer.

Synthesis of ATAOPV

Compound **A** was synthesized as detailed in reference S1 and S2.

Synthesis of [4-(2-{2,5-dimethoxy-4-[2-(4-nitro-phenyl)-vinyl]-phenyl}-vinyl)-phenyl]-trimethyl-ammonium; iodide (**B**)

Compound **A** (0.5 g, 1.24 mmol) and sodium bicarbonate (1.0 g, 12.4 mmol) were dissolved in 10 ml DMF and purged with nitrogen gas. Iodomethane (0.69 ml, 7.44 mmol) was dropped into the reaction and the mixture was allowed to stir at 40 ℃ for 24 h. The solution was cooled and added excess acetone to remove sodium bicarbonate by precipitation. The mixture was filtered and the residue was purified by recrystallization with toluene. The product is an orange product (0.6 g, yield: 85 %). ^{1}H NMR (400 MHz, DMSO): δ 3.61 (s, 9H), 3.92 (s, 6H), 7.38-7.41 (d, 2H), 7.41-7.67 (m, 4H), 7.80-7.84 (m, 4H), 7.94-7.97 (d, 2H), 8.21-8.24 (d, 2H).

Synthesis of [4-(2-{4-[2-(4-amino-phenyl)-vinyl]-2,5-dimethoxy-phenyl}-vinyl)-phenyl]-trimethyl-ammonium; iodide (**ATAOPV**)

Compound **B** (0.1 g, 0.174 mmol) was dissolved in anhydrous ethanol and purged with nitrogen gas. 40% stannous chloride (0.18 ml) was dropped slowly and allowed to stir at 70 ^{o}C for 18 h. The solution was cooled and filtered. The remained powerer was dissolved in methanol again and triethylamine was added slowly to basify the solution. Solution color was changed from orange to yellow and purified by recrystallization with acetone. The mixture was filtered and the product is a yellow product (70 mg, yield: 74 %). ^{1}H NMR (400 MHz, DMSO): δ 3.60 (s, 9H), 3.87-3.89 (d, 6H), 5.34 (s, 2H), 6.55-6.57 (d, 2H), 7.07-7.25 (m, 2H), 7.26-7.55 (m, 6H), 7.77-7.79 (d, 2H), 7.92-7.94 (d, 2H); ^{13}C NMR (400 MHz, DMSO) δ(ppm) 56.8, 98.7, 109.2, 109.9, 113.3, 116.2, 121.3, 124.4, 125.9, 126.6, 127.7, 127.8, 128.1, 130.3, 139.8, 146.2, 151.0, 151.8; FT-IR (KBr, cm^{-1}): 3431, 3204, 3043, 2829, 1626, 1592, 1515, 1492, 1464, 1409, 1345, 1327, 1262, 1208, 1178, 1124, 1044, 962, 853, 815, 682, 557, 515; mass spectrum EI calcd m/z of [M] = 415.24, measured m/z of [M] = 415.32.

Reference

S1. Y. I. Park, C. Kuo, J. S. Martinez, Y. Park, O. Postupna, A. Zhugayevych, S. Kim, J. Park, S. Tretiak, and H. Wang, *ACS Appl. Mater. Interfaces* 5, (2013) 4685-4695.

S2. U. Caruso, M. Casalboni, A. Fort, M. Fusco, B. Panunzi, A. Quatela, A. Roviello, F. Sarcinelli, *Optical Materials* 27 (2005) 1800–1810.

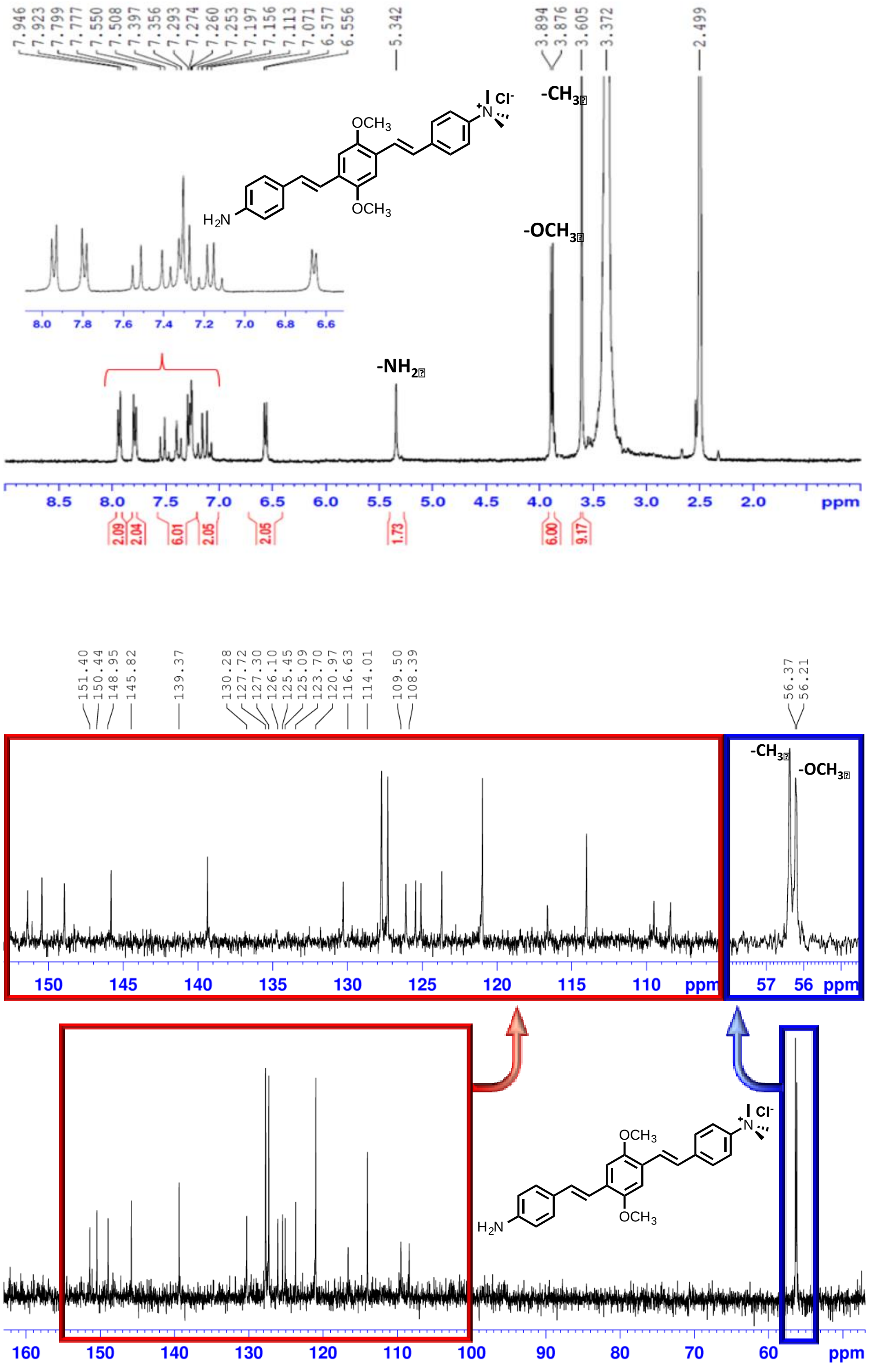

Figure S1. ^{1}H and ^{13}C NMR of **ATAOPV**.

Chromatogram

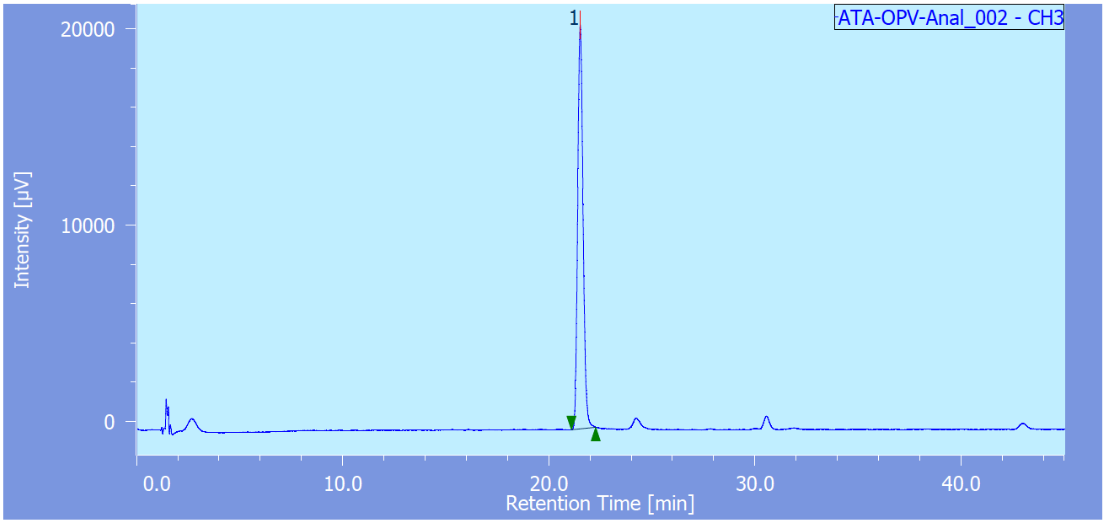

Chromatogram Information	
User Name	hplc
Date Modified	8/4/2014 3:18:43 PM
Description	
HPLC System Name	HPLC
Injection Date	8/4/2014 2:33:42 PM
Volume	10.00 [µL]
Sample Number	1
Project Name	Analytical Data
Acquisition Time	45.0 [min]
Acquisition Sequence	
Control Method	ATA_OPV_anal
Peak ID Table	
Calibration Method	
Additional Information	

Channel & Peak Information Table	
Chromatogram Name	ATA-OPV-Anal_002-CH3
Sample Name	ATA-OPN-anal
Channel Name	UV3
Sampling Interval	500 [msec]
Peak Method	(Manual)
Formula	
Decision	

#	Peak Name	CH	tR [min]	Area [µV·sec]	Height [µV]	Area%	Height%	Quantity	NTP	Resolution	Symmetry Factor	Warning
1	Unknown	3	21.525	374127	20574	100.000	100.000	N/A	32676	N/A	1.149	

Figure S2. HPLC chromatogram of **ATAOPV**.

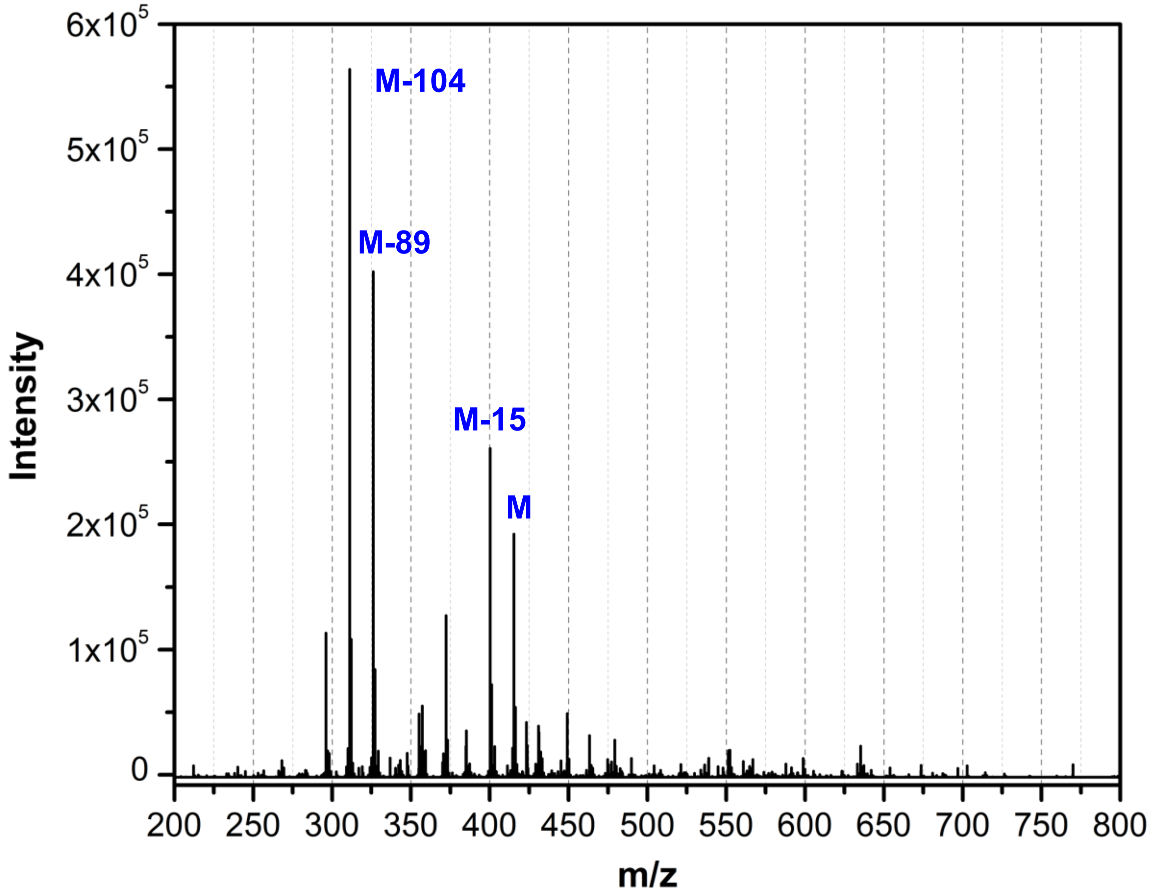

Figure S3. ESI-MS spectrum of **ATAOPV**.

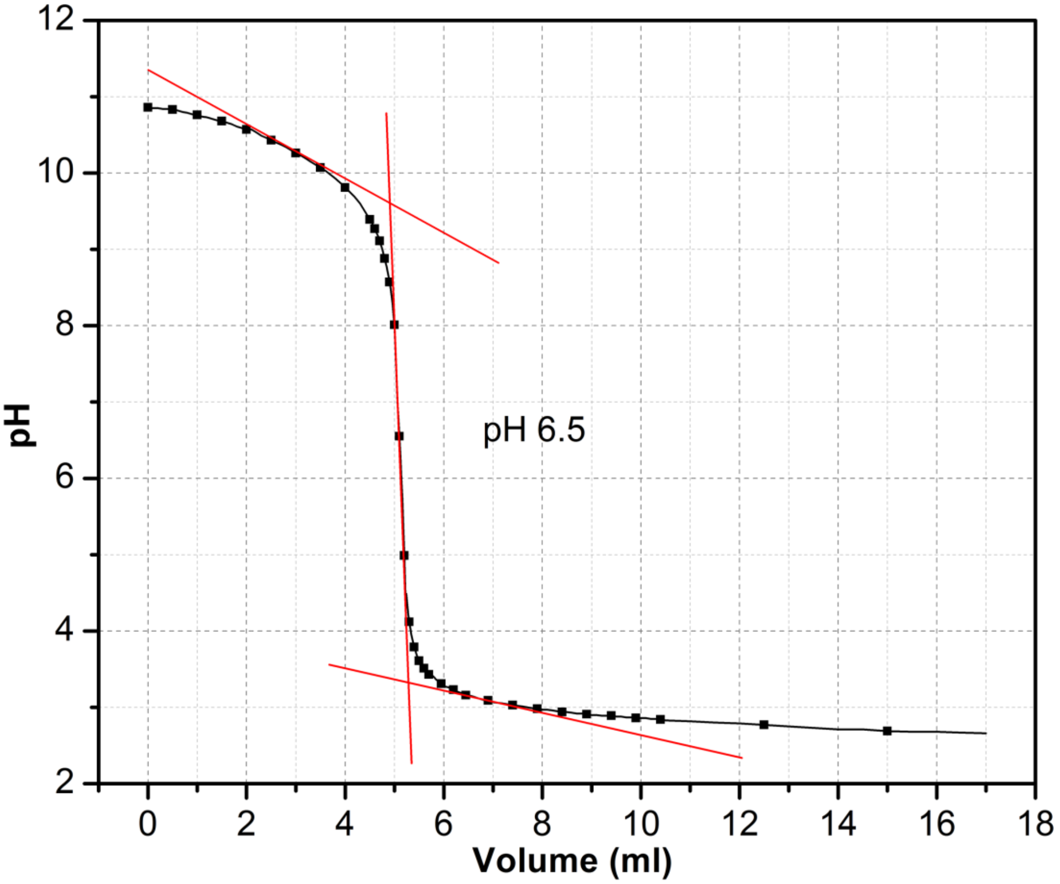

Figure S4. Titration curve of **ATAOPV**.

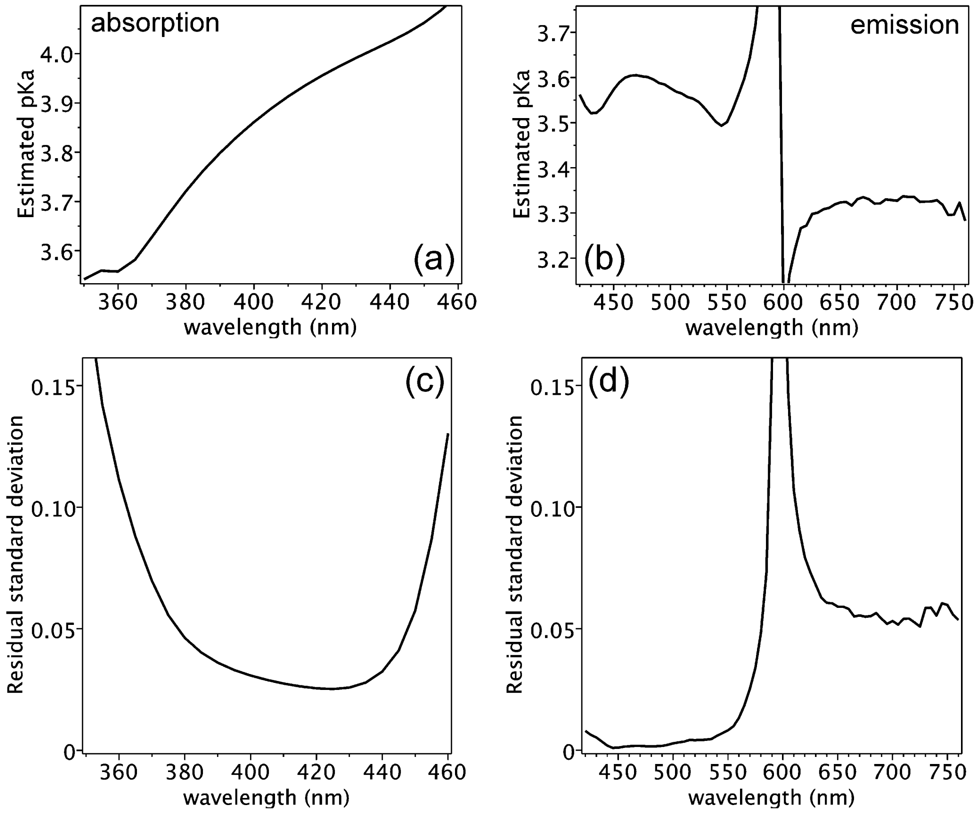

Figure S5. Estimation of pK_a from absorption/emission spectra.

Calculation of acid dissociation constant

Based on Born-Haber cycle, Gibbs free energy change of the dissociation reaction in solution ($AH_{sol} = A^-_{sol} + H^+_{sol}$), denoted as ΔG_{sol}, can be evaluated as follows:[1]

$$\Delta G_{sol} = \Delta G_{gas}(AH) - \Delta G_{sol}(AH) + \Delta G_{sol}(A^-) + \Delta G_{sol}(H^+),$$

$$\Delta G_{gas}(AH) = G_{gas}(A^-) + G_{gas}(H^+) - G_{gas}(AH).$$

Here AH corresponds to protonated molecule and A^- to non-protonated one. Gas-phase free energies of AH and A^- were computed in vacuum. Since the electronic energy of the proton is zero, its gas-phase free energy is derived from a *PV* enthalpy term and a translational free energy, and for 298 K at 1 atm is usually set to -26.28 kJ/mol.[1,2] Change in solvation energy of the proton, $\Delta G_{sol}(H^+)$, is known from experiment, and equals -1104.5 kJ/mol.[3] This value takes into account standard state correction energy, equal to 7.91 kJ/mol. Acid dissociation constant (K_a) can be calculated from ΔG_{sol}:

$$pK_a = \Delta G_{sol}/RT\ln 10,$$

where R is the universal gas constant, T is temperature.

For the given system, ΔG_{sol} was found to equal 35 kJ/mol. This gives pK_a = 6.2, which suggests that the molecule acts as a weak acid. A simple conversion from pK_a to pH was made in order to relate molecular processes to the acidity of the media, using the ICE chart. Corresponding pH was found to be 5.6.

[1] C. Cramer, Essentials of computational chemistry, Wiley (2004), p.412.
[2] D. Jacquemin, E. A. Perpete, I. Ciofini, C. Adamo, J. Phys. Chem. A 112, 794 (2008).
[3] M. D. Tissandier, K. A. Cowen, W. Y. Feng *et al.*, J. Phys. Chem. A 102, 7787 (1998).

Estimation of pK_a from the experimental data

To estimate pK_a from pH-dependence of the absorption and emission spectra (see Fig. 2), the latter were assumed to be a linear superposition of spectra of two molecular species (protonated and non-protonated):

$$I_{observed}(\mathrm{pH}, \lambda) = N_1(\mathrm{pH})I_1(\lambda) + N_2(\mathrm{pH})I_2(\lambda) \tag{S1}$$

where λ is the wavelength, $N_{1,2}$ is the number of molecules of each kind, and $I_{1,2}$ is the absorption/emission intensity of one molecule. The ratio N_1/N_2 was approximated by the formula (1). At each λ the experimental spectrum was smoothed by 4-th order polynomial fit to the experimental data in the range from λ-10 nm to λ+10 nm. Thus obtained set of $I_{observed}$(pH,λ) was normalized to [0,1] range and fitted by the formula (S1) using three parameters: pK_a and normalized $I_{1,2}$. The results are shown in Fig. S5. Spectral regions with $I_1 \approx I_2$ have large fitting error. Therefore they are excluded from the analysis. The rest of the spectra consist of three regions with fitting errors small enough for estimating pK_a: absorption spectrum near the main absorption peak (350-460 nm), emission spectrum near the blue PL peak (430-570 nm), and emission spectrum near the red PL peak (630-760 nm).

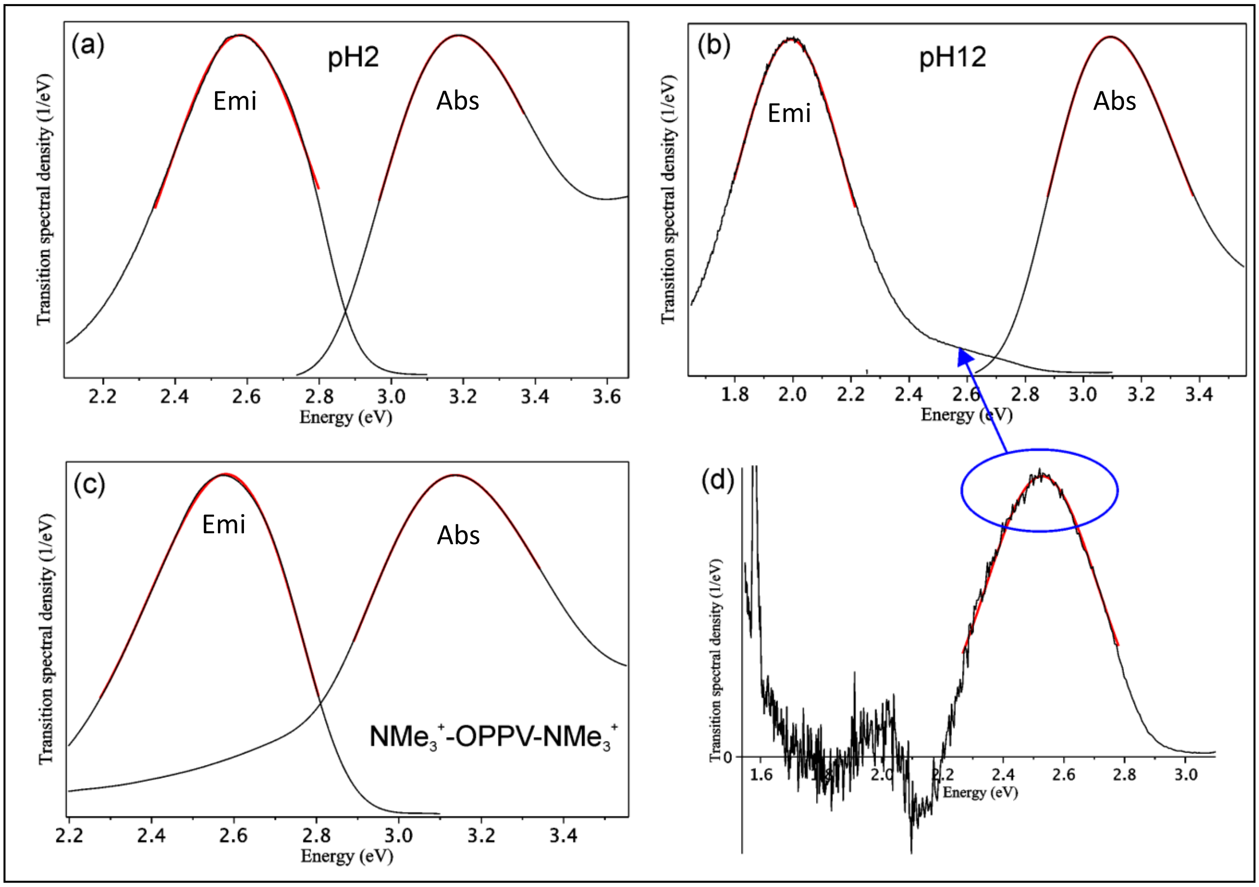

		lg OS	E_{exc} (eV)	σ_E (eV)	$\hbar\omega_Q$ (eV)	S_Q
(a)	pH 2, absorption	-2.242(1)	3.264(4)	0.237(1)	0.21(0)	0.8(0)
(b)	pH 12, absorption	-1.995(9)	3.164(8)	0.234(9)	0.21(4)	0.7(2)
(a)	pH 2, emission	4.92(1)	2.58(3)	0.20(0)	—	—
(b)	pH 12, emission	3.82(2)	1.99(3)	0.19(3)	0	0
(d)	pH 12, emission, blue component	2.65	2.53	0.19	0	0
(c)	NMe_3^+-OPV-NMe_3^+, absorption		3.21(3)	0.26(3)	0.28(11)	0.4(1)
(c)	NMe_3^+-OPV-NMe_3^+, emission		2.527(7)	0.187(0)	0.14(1)	1.3(1)

Figure S6. Single quantum mode fit to experimental spectra at pH 2 and pH 12. The fitting parameters are given in the table together with NMe_3^+-OPV-NMe_3^+ in water for comparison. The logarithm of the oscillator strength, lg OS, is meaningful only if taken as difference within the same dataset (absorption or emission). For the emission spectra at pH 2 there are systematic deviations from a single quantum mode lineshape (at least two gaussian-shaped components are needed). The blue emission at pH 12 is processed after the subtraction of the best-fit single quantum mode lineshape of the main PL band, the residual signal is shown in panel (d).

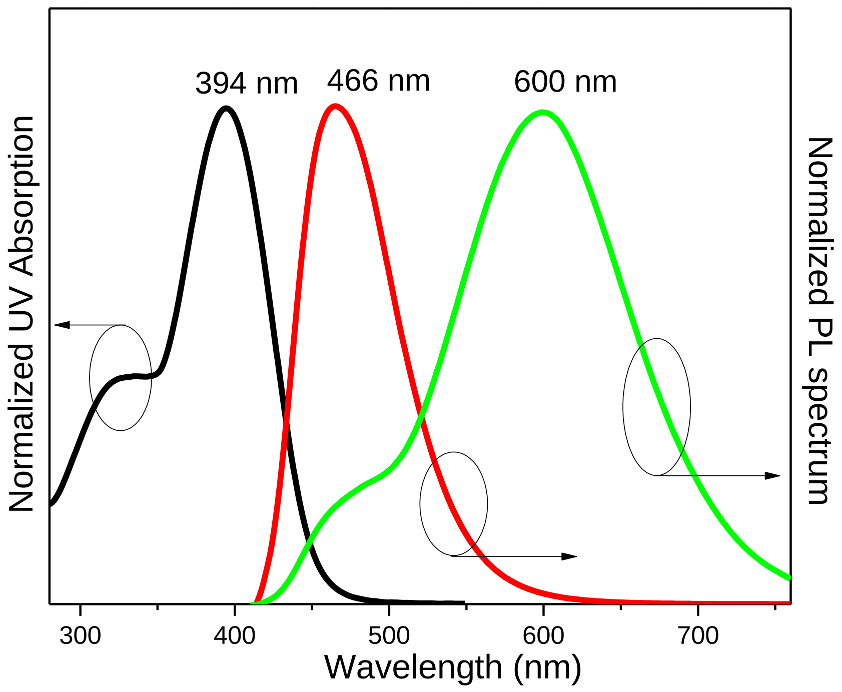

Figure S7. UV/Vis absorption of **ATAOPV** in pH 12 (Black) and PL spectra (Red) in pH 2 and (Green) in pH 12.

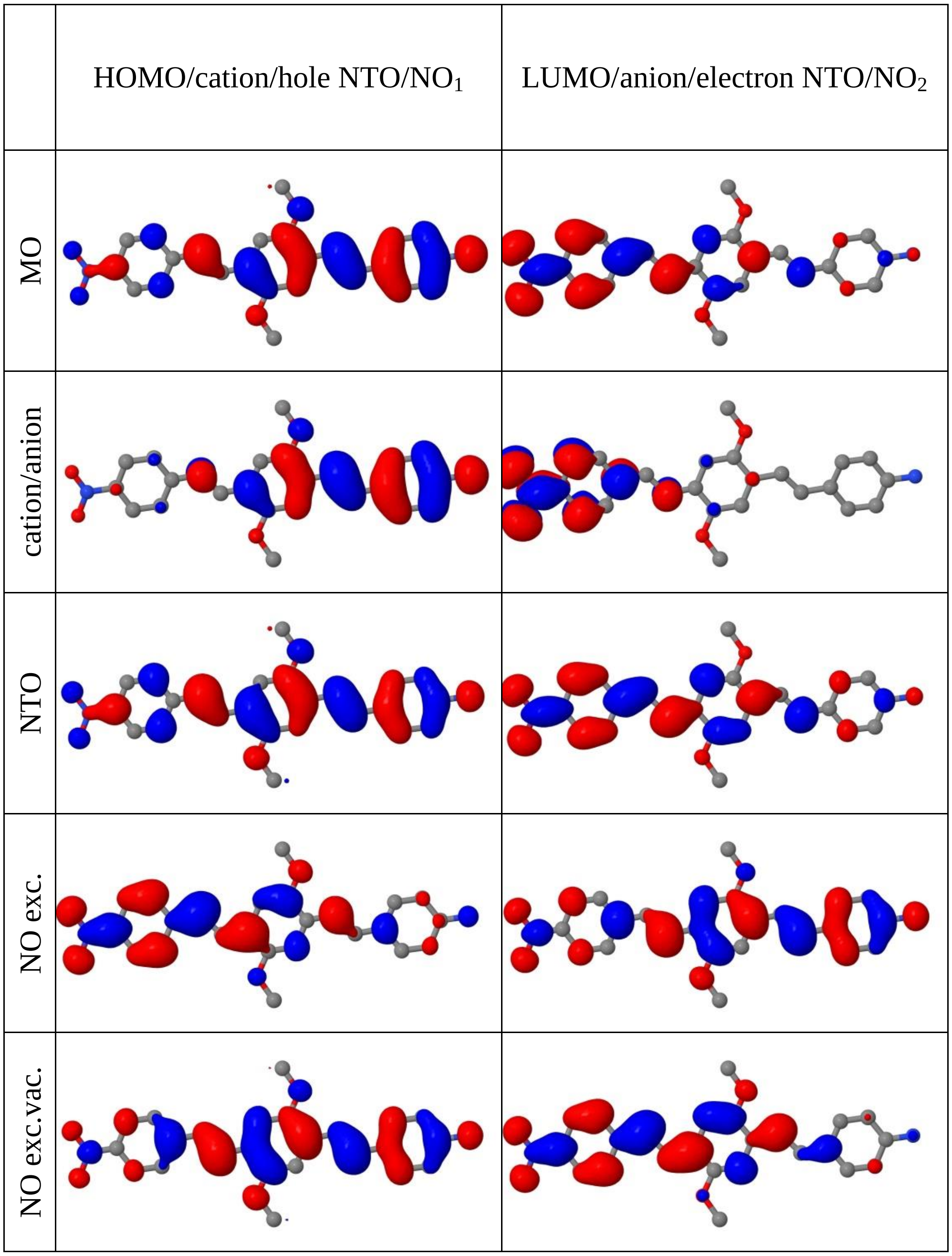

Figure S8. Comparison of different orbitals for molecule **A** in DMSO, see Fig. S10 for explanation. NO exc. occupations are 1.12 and 0.89, NO exc.vac. occupations are 1.24 and 0.78. Note the HOMO-LUMO mixing for "NO exc." set of orbitals. Compared to the molecules shown in Fig. 5 OS_{abs} = 1.1, λ_{abs} = 513 nm, λ_{emi} = 676 nm, τ_{rad} = 2.6 ns, $\Delta\lambda$ = +206 nm, Δq = -0.40, E_{CT} = -0.75 eV.

		Hole NTO	Electron NTO
OPV-NH_2	OS_{abs} = 1.7<br>λ_{abs} = 364 nm<br>λ_{emi} = 473 nm<br>τ_{rad} = 1.3 ns<br>$\Delta\lambda$ = +38 nm<br>E_{CT} = 0.04 eV	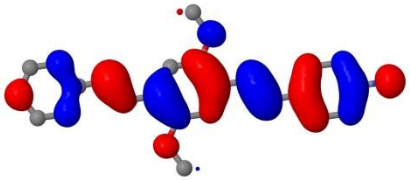	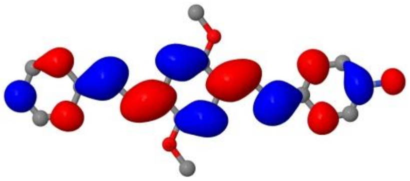
Non-protonated	OS_{abs} = 1.7<br>λ_{abs} = 379 nm<br>λ_{emi} = 474 nm<br>τ_{rad} = 1.2 ns<br>$\Delta\lambda$ = -27 nm<br>E_{CT} = -0.07 eV	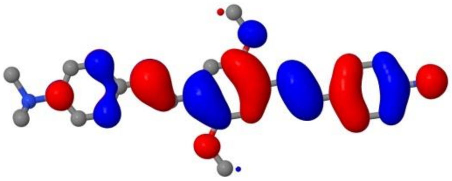	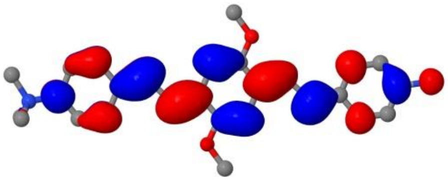
Protonated	OS_{abs} = 1.5<br>λ_{abs} = 374 nm<br>λ_{emi} = 446 nm<br>τ_{rad} = 1.3 ns<br>$\Delta\lambda$ = -16 nm<br>E_{CT} = 0.10 eV	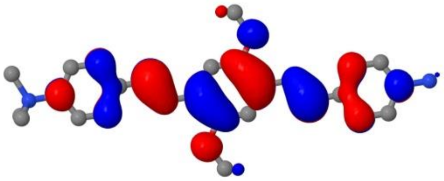	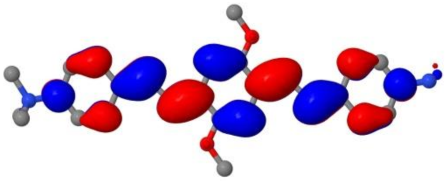
OPV	OS_{abs} = 1.4<br>λ_{abs} = 357 nm<br>λ_{emi} = 439 nm<br>τ_{rad} = 1.3 ns<br>$\Delta\lambda$ = +16 nm<br>E_{CT} = 0.15 eV	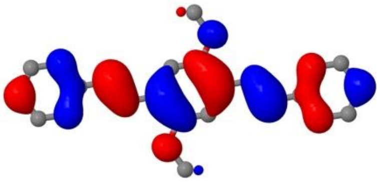	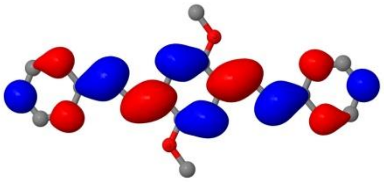

Figure S9. (Extended version of **Figure 5**) Calculated properties of the first excited state in water of protonated and non-protonated molecules as well as their simplified π-isoelectronic analogues. Natural transition orbitals are shown for the lowest excitation in the relaxed geometry. OS is oscillator strength, τ_{rad} is radiative lifetime, $\Delta\lambda=\lambda_{emi}(water)-\lambda_{emi}(vacuum)$ is solvatochromic shift, $E_{CT} = E_{cation}+E_{anion}-E_{exciton}-E_{ground}$ is the intermolecular CT energy relative to intramolecular exciton (relaxed geometries, equilibrium solvation).

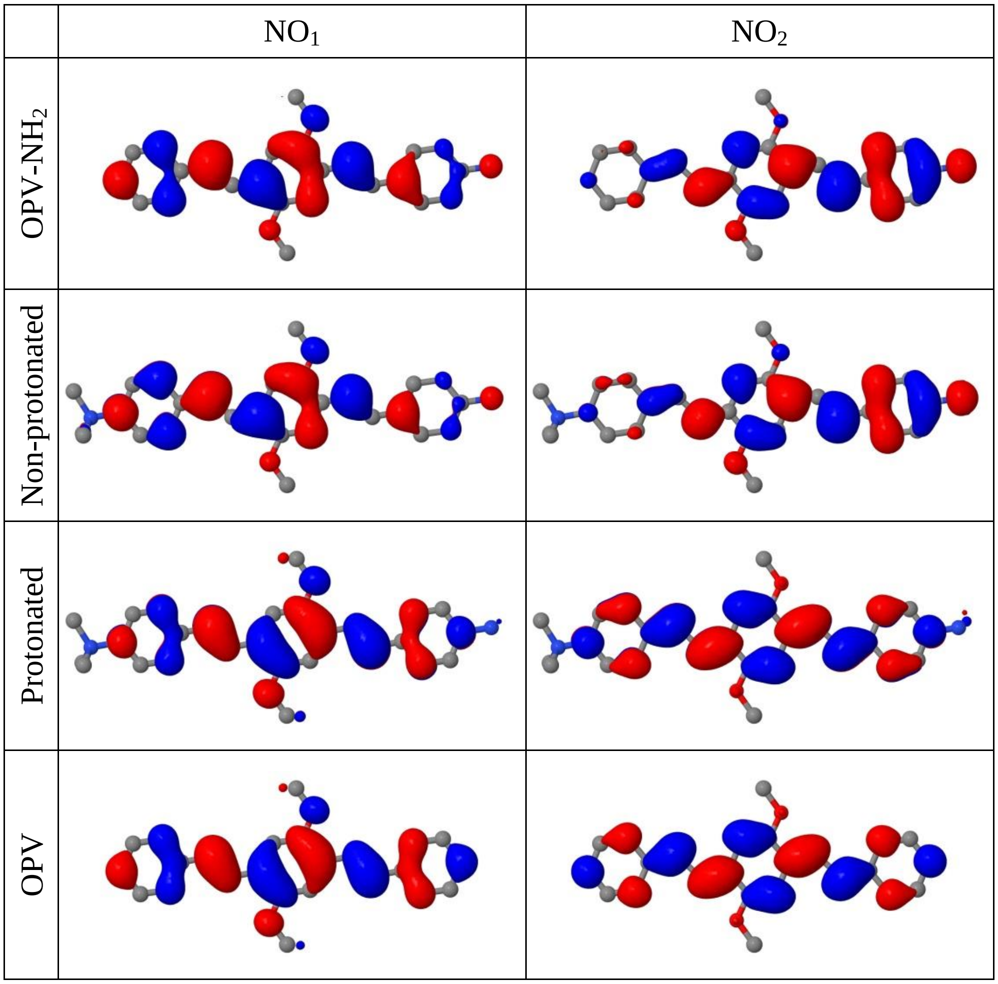

Figure S10. Half-occupied natural orbitals of the lowest excited state in the relaxed geometry (ordered by the occupation number).

	Cation	Anion
OPV-NH_2	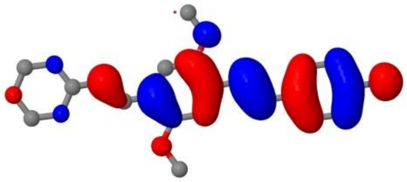	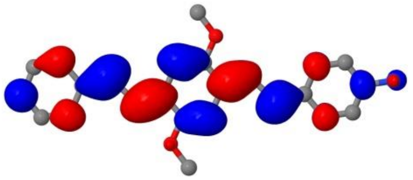
Non-protonated	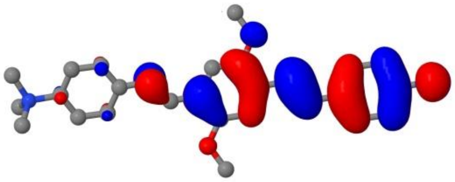	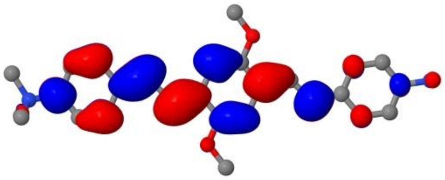
Protonated	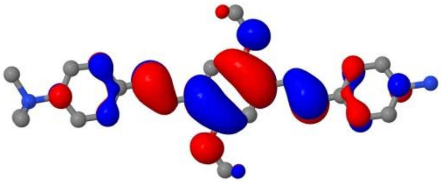	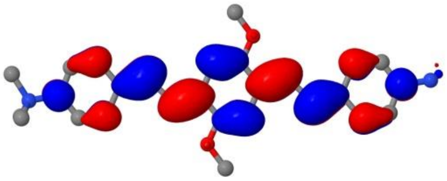
OPV	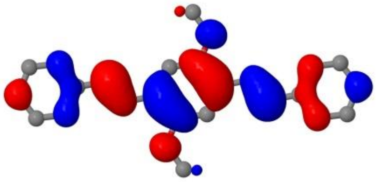	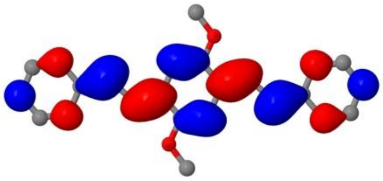

Figure S11. Half-occupied natural orbitals of the cation and anion in the relaxed geometry.

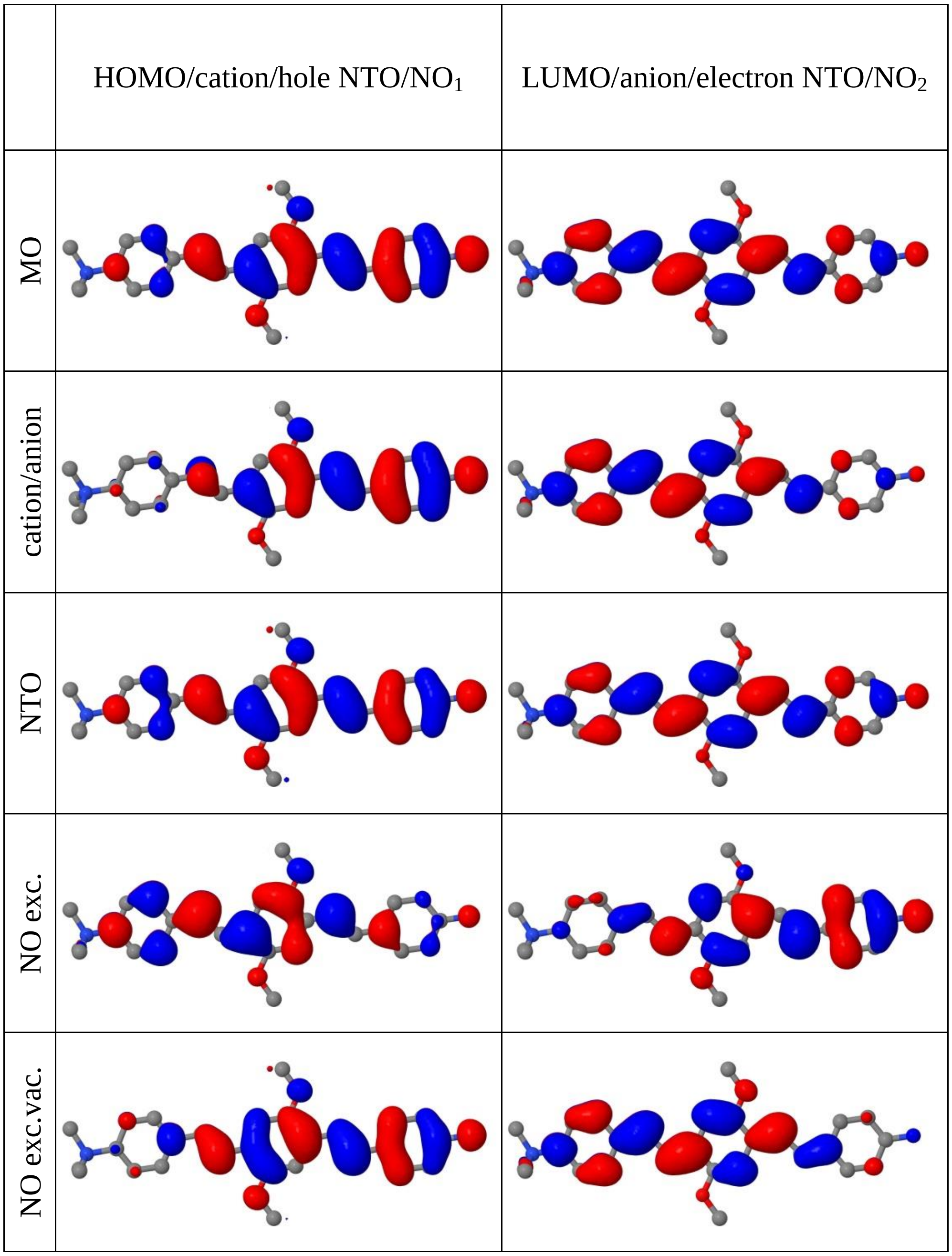

Figure S12. Comparison of different orbitals for non-protonated molecule in water: MO – molecular orbitals HOMO/LUMO, cation/anion – half-occupied natural orbitals of the cation and anion, NTO – natural transition orbitals of the lowest excitation, NO exc. – half-occupied natural orbitals of the lowest excited state, NO exc.vac. – the same in vacuum. The geometry is relaxed in the lowest excited state except for cation and anion. Note the HOMO-LUMO mixing for "NO exc." set of orbitals.

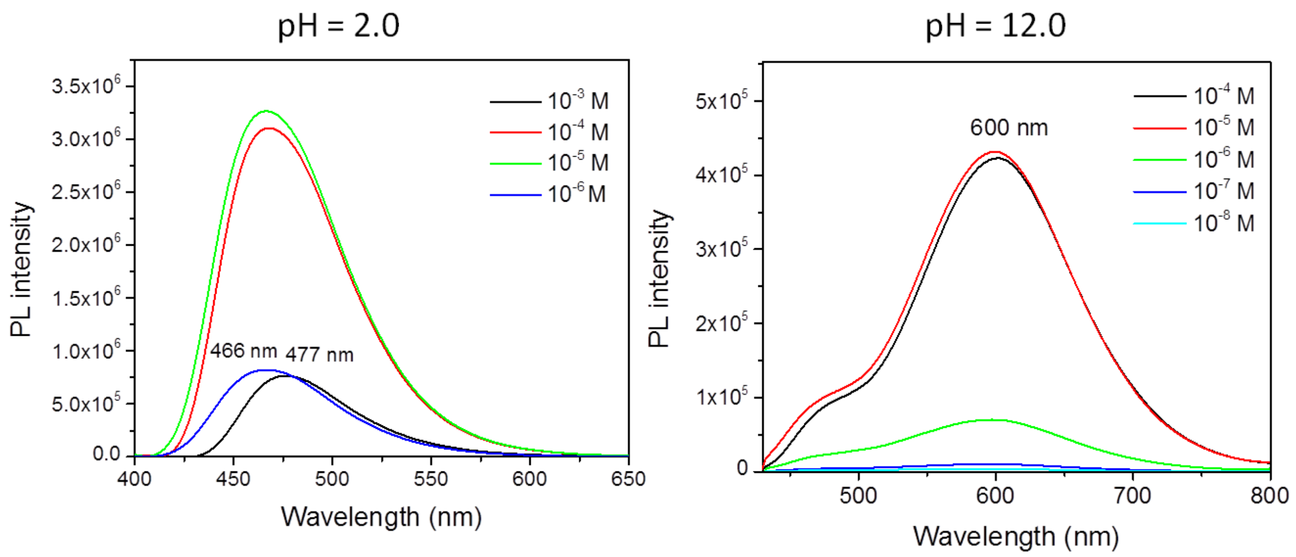

Figure S13. Measured PL spectra of **ATAOPV** at different concentrations under different pH.

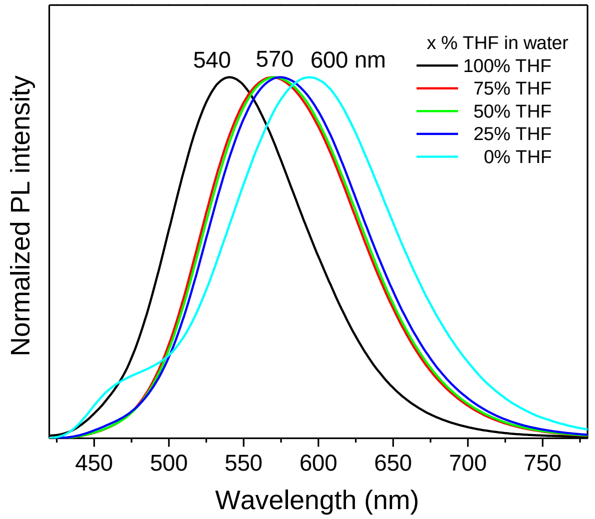

Figure S14. PL spectra of ATAOPV in various solvents and solvent combinations.

(a)

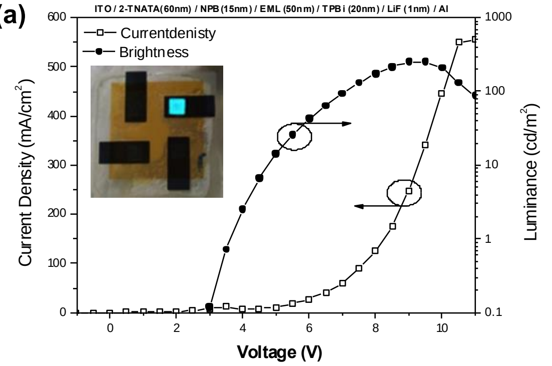

Voltage	Current density (A/cm^2)	Luminance (cd/m^2)	L.E (cd/A)	P.E (lm/W)	Q.E (%)	C.I.E. (x,y)
6	27.9	42.49	0.152	0.0882	0.0784	(0.2096, 0.3352)
7	59.7	93.9	0.157	0.0781	0.0847	(0.2045, 0.3166)
8	125	172.4	0.138	0.0599	0.0780	(0.2003, 0.2986)
9	247	251	0.102	0.0392	0.0606	(0.1972, 0.2812)

(b)

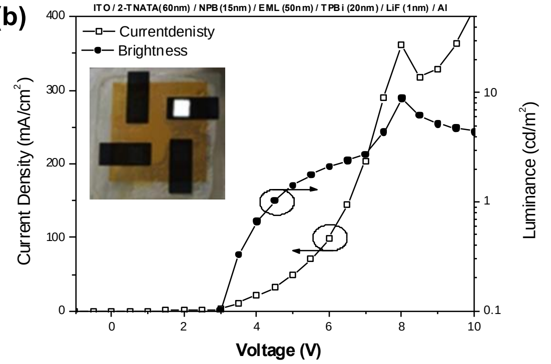

Voltage	Current density (A/cm^2)	Luminance (cd/m^2)	L.E (cd/A)	P.E (lm/W)	Q.E (%)	C.I.E. (x,y)
6	98.8	2.11	0.00214	0.00124	0.00109	(0.2965, 0.3924)
7	204	2.73	0.00134	0.00066	0.00076	(0.3223, 0.3838)
8	361	8.85	0.00245	0.00106	0.00126	(0.3402, 0.4315)
9	329	5.16	0.00157	0.00061	0.00089	(0.3134, 0.3822)

(c)

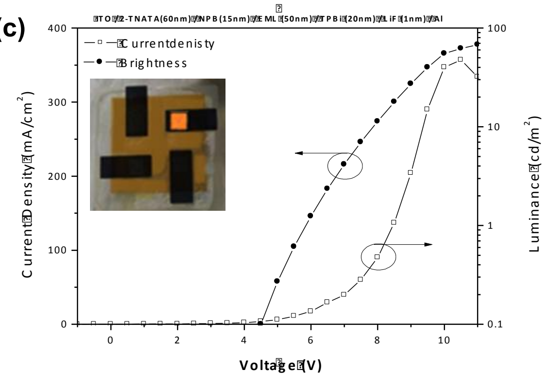

Voltage	Current density (A/cm^2)	Luminance (cd/m^2)	L.E (cd/A)	P.E (lm/W)	Q.E (%)	C.I.E. (x,y)
7	39.5	4.15	0.0105	0.00521	0.00507	(0.4614, 0.4798)
8	90.3	11.34	0.0126	0.00545	0.00596	(0.4552, 0.4808)
9	204	27.2	0.0133	0.00515	0.0062	(0.4455, 0.4818)
10	347	55.19	0.0159	0.00553	0.00697	(0.4008, 0.4823)

Figure S15. Current density−voltage and luminance−voltage characteristics of (a) blue, (b) white, and (c) orange EL devices.

Table S1. Experimental and calculated absorption and emission excitation energies. The basis set is 6-31G* except for "Non-protonated+I^-" where CEP-31G basis set is used. As expected[4] the calculated IP+HOMO (IP=Ionization Potential) in vacuum correlate well with excitation energy errors. Dimer geometries are not optimized unless "opt." is present. Selected dimer configurations: 1 – molecules are in ground/excited state geometry separated by 3.5 Å, 2 – molecules are in ground/cation+anion geometries separated by 3.5 Å. In "separated charges" configuration the absorption is taken as single molecule process and emission is calculated as E(+1,+1)–E(0,+1)+E(–1,–1)–E(0,–1), where $E(Q_1,Q_2)$ is the energy of a molecule with extra charge Q_1 in the geometry optimized with charge Q_2.

System	Method	IP+HOMO eV	Absorption LR, eV	Absorption SS, eV	Emission SS, eV	Stokes shift SS, eV
Protonated species in water						
pH 2	Experiment		3.26		2.58	0.68
Single molecule	CAM-B3LYP	+0.26	3.25	3.32	2.78	0.54
	ωB97X	–0.52	3.56	3.62	2.90	0.72
	B3LYP	+1.25	2.71	2.76	2.45	0.31
Non-protonated species in water						
pH 12	Experiment		3.16		1.99	1.17
Single molecule	CAM-B3LYP	+0.33	3.22	3.28	2.62	0.66
	ωB97X	–0.40	3.56	3.62	2.79	0.83
	B3LYP	+1.21	2.59	2.53	2.27	0.26
Molecule + I^-	CAM-B3LYP		3.05	3.10	2.46	0.64
Molecule + Cl^-	CAM-B3LYP		3.23	3.29	2.54	0.75
Dimer config. 1 opt.	ωB97X		3.58	3.67	—	—
Dimer config. 1	CAM-B3LYP		3.16	3.22	2.16	1.06
Dimer config. 2	CAM-B3LYP		3.16	3.22	1.74	1.48
Separated charges	CAM-B3LYP		3.22	3.28	2.12	1.16
Non-protonated species in tetrahydrofuran						
	Experiment		2.98		2.19	0.79
Single molecule	CAM-B3LYP		3.16	3.28	2.60	0.68
Separated charges	CAM-B3LYP		3.16	3.28	2.38	0.90
Other molecules in water						
	Experiment		3.21		2.53	0.68
NMe_3^+-OPV-NMe_3^+	CAM-B3LYP	+0.25	3.24	3.30	2.78	0.52
	ωB97X	–0.53	3.56	3.62	2.90	0.72
	B3LYP	+1.23	2.70	2.74	2.43	0.31

[4] R. Baer, E. Livshits, U. Salzner, Annu. Rev. Phys. Chem. 61, 85 (2010).
